# Magnetic microrobots for precision medicine: From locomotion mechanisms and advanced materials to clinical translation

**DOI:** 10.1002/btm2.70197

**Published:** 2026-11-08

**Authors:** Gongxin Li, Yuting Mao, Fei Liu, Fangmei An, Qiang Zhan

**Affiliations:** ^1^ The Key Laboratory of Advanced Process Control for Light Industry (Ministry of Education), Institute of Automation Jiangnan University Wuxi China; ^2^ Department of Gastroenterology The Affiliated Wuxi People's Hospital of Nanjing Medical University Wuxi Jiangsu China

**Keywords:** advanced materials, magnetic field‐driven, microrobots, minimally invasive medicine

## Abstract

As minimally invasive surgery (MIS) ventures into increasingly confined and delicate anatomical regions, conventional tethered instruments encounter inherent limitations in miniaturization and maneuverability. Untethered magnetic microrobots, characterized by precise navigation, deep tissue penetration, and highly efficient propulsion in low‐Reynolds‐number fluids, have emerged as a transformative platform for targeted diagnostics and therapeutics with sub‐millimeter accuracy. This review systematically examines the state‐of‐the‐art breakthroughs and the clinical translation landscape of magnetic microrobotic systems. Specifically, we first categorize and analyze four distinct propulsion modalities—translation, swimming, tumbling, and jumping—alongside their adaptive strategies for navigating unstructured physiological environments. Subsequently, the core technological framework is detailed, encompassing material selection (ranging from functional to bio‐integrated materials) and cross‐scale fabrication techniques such as two‐photon polymerization and microfluidics. Finally, the discussion underscores recent milestones in vascular interventions, gastrointestinal diagnostics, and oncology, while exploring emerging frontiers such as ophthalmic surgery and tissue engineering.

**Translational Impact Statement**

Magnetic microrobots offer opportunities for targeted therapy and minimally invasive intervention in anatomically confined regions. This review connects locomotion requirements with material selection, fabrication strategies, and biomedical applications to guide the development of clinically relevant systems. By comparing actuation conditions, locomotion performance, and drug‐release strategies, it supports the selection of robotic platforms for different physiological environments. It also identifies biocompatibility, image‐guided navigation, scalable manufacturing, and validation under realistic physiological conditions as key priorities for advancing magnetic microrobots toward clinical translation.

## 1 INTRODUCTION

Advances in minimally invasive surgery (MIS) have profoundly transformed modern clinical practice by minimizing surgical trauma. However, when dealing with lesions located deep within the body, in structurally complex areas, or in highly sensitive regions, existing access tools—such as catheters and endoscopes—still face severe physical limitations. Constrained by the physical dimensions, mechanical stiffness, and friction with blood vessel walls of these instruments, traditional tools often struggle to penetrate sub‐millimeter‐scale and highly tortuous vascular networks, making it impossible to reach distal lesions.^1^ In addition, current drug therapies primarily rely on systemic circulation for delivery; this approach not only results in low bioavailability but also often leads to severe systemic toxic side effects due to the non‐specific distribution of drugs in non‐target tissues.^2^ Therefore, there is an urgent clinical need for a tether‐free, miniature, and highly flexible microcarrier capable of precisely reaching deep tissues and delivering targeted therapy.^3^ A recent review has further highlighted the potential of magnetically guided microrobots for targeted drug delivery and clinical translation, with particular emphasis on magnetic navigation, payload release, biosafety, and therapeutic efficacy.^4^

Microrobots typically refer to miniature devices ranging in size from micrometers to millimeters that can convert magnetic, optical, acoustic, or chemical energy into mechanical energy, thereby enabling controlled movement in fluid environments.^5^ Due to their ultra‐compact size and agile maneuverability, microrobots can overcome the physical size limitations of traditional catheters and endoscopes, penetrating narrow and complex physiological environments such as the body's microvasculature, bile ducts, or the brain; they are regarded as the ideal vehicles for achieving precision medicine.^6^ Among the various propulsion strategies for microrobots, magnetic field propulsion stands out as an ideal solution to these challenges due to its unique physical properties. Compared to optical, thermal, or electric field propulsion, low‐frequency magnetic fields are virtually transparent to biological tissue, enabling safe penetration into deep tissues for remote control without significant thermal damage. Moreover, magnetic materials typically exhibit good biocompatibility.^7^ More importantly, in fluid environments at the micrometer scale with low Reynolds numbers, the magnetic torque generated by a rotating magnetic field can effectively overcome viscous drag, enabling the robot to achieve efficient propulsion—an advantage that cannot be matched by propulsion relying solely on gradient forces.^8^ By precisely adjusting the frequency, amplitude, and direction of an external magnetic field, soft magnetic robots can even flexibly switch between various movement modes—such as tumbling, jumping, and swimming—in response to environmental changes, thereby adapting to the complex, unstructured terrain inside the human body.

In recent years, research on magnetic microrobots has shifted from exploring single‐function motion mechanisms toward multifunctional integration and clinical applications. For example, recent studies have demonstrated magnetic microrobot systems for on‐demand gastric drug delivery, biodegradable microrobots with adjustable locomotion, and adaptive navigation in unstructured dynamic environments.^9^, ^10^, ^11^ Researchers have not only developed multifunctional capsule robots capable of performing complex tasks such as targeted drug delivery and liquid biopsy sampling, but have also achieved anti‐scouring navigation and embolization therapy by using magnetic fields to control swarms of micro‐ and nanoparticles within dynamic blood flow.^8^ With the successful validation of the relevant technology in live animal models, selecting the most appropriate movement strategy for different physiological environments has become crucial.^2^ Therefore, this review conducted a search using keywords such as “microrobots, advanced materials, magnetic field‐driven, minimally invasive medicine” in databases such as Web of Science, compiling a total of 121 papers. Unlike conventional materials‐oriented reviews, this review adopts a function‐driven framework that prioritizes locomotion as the fundamental requirement for magnetic microrobots to navigate complex physiological environments and reach targeted sites. Accordingly, locomotion mechanisms are discussed first, followed by the material selection and fabrication techniques required to realize these motion capabilities, and finally their biomedical applications and clinical translation. This organization establishes a logical progression from functional requirements to enabling technologies and clinical implementation, as outlined in Figure 1.

**FIGURE 1:**
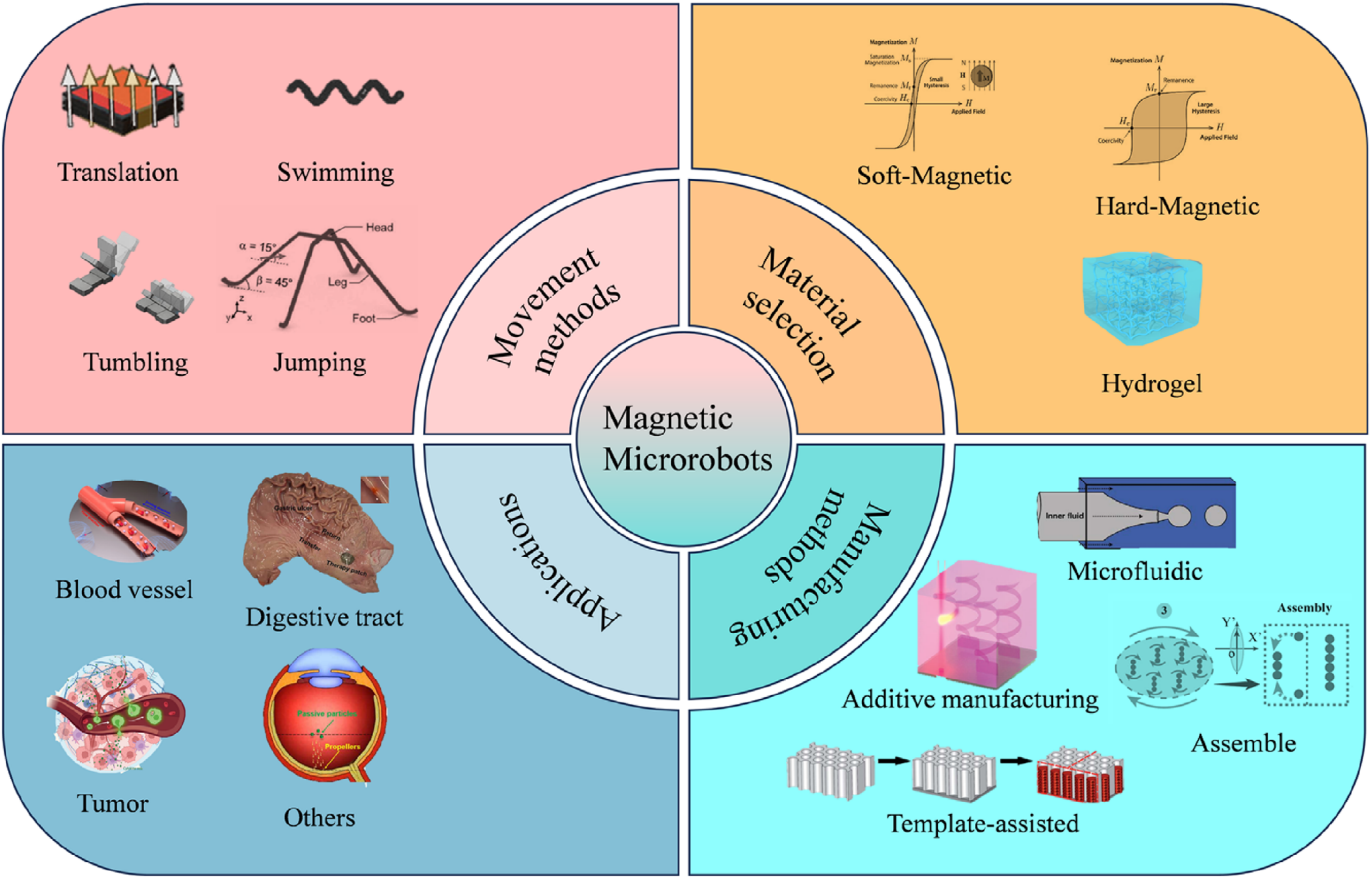
The schematic diagram of magnetic microrobots covers four aspects: motion, materials, manufacturing, and applications.

## 2 MAGNETIC MICROROBOT MOTION MODES

The efficient motion of magnetic microrobots is fundamental to achieving precise navigation and operations within complex physiological environments. Due to the highly unstructured nature of the human internal environment, which encompasses everything from low‐Reynolds‐number Newtonian/non‐Newtonian fluids (such as blood and vitreous humor) to tissue surfaces with varying friction coefficients and topographies (such as blood vessel walls and gastric mucosa), a single locomotion strategy often struggles to address all operational conditions. Therefore, leveraging magnetic forces and moments generated by external magnetic fields, combined with the structural design and material properties of the microrobots themselves, researchers have developed various biomimetic or non‐biomimetic motion modes to overcome the effects of microscopic forces such as fluid resistance and surface friction.^12^

This section categorizes the motion patterns of microrobots into four types—translation, swimming, tumbling, and jumping—based on their kinematic characteristics and interaction methods with environmental media. It further discusses the driving mechanisms, control methods, and limitations of each mode (Table 1). Representative robot dimensions, magnetic‐field conditions, and quantitative locomotion performance are summarized in Table 2 to facilitate comparison across the four motion modes.

**TABLE 1:** Comparison of motion modes of magnetically controlled microrobots.

Motion modes	Core driving mechanism	Control methods	Advantages	Limitations	References
Translation	Magnetic gradient force; torque‐assisted stick–slip actuation can generate translation at solid interfaces	Regulation of magnetic‐field gradient magnitude and direction	Simple mechanism, group convergence and towing	Requires a magnetic field generator capable of producing high gradients; highly susceptible to friction forces; difficult to maintain group consistency	13, 14, 15, 16, 17, 18, 19
Swimming	Rotating or oscillating magnetic fields	Regulation of field amplitude, frequency, rotation axis, phase, and rotation direction	Helical morphology facilitates upstream locomotion; proven efficacy for drug/cell transport; excellent biocompatibility	Requires precision spiral or flexible tail structure; weak surface adhesion; limited load capacity	21, 22, 23, 24, 25, 26, 27, 28
Tumbling	Rotating magnetic field‐induced torque coupled with surface friction	Regulation of field‐rotation plane, direction, amplitude, and frequency	Strong traction; precise operations on tissue surfaces; capable of end‐to‐end tumbling to adapt to rough terrain	Prone to idling on low‐friction or excessively lubricated surfaces; strictly requires continuous contact with the anatomical base	22, 29, 30, 31
Jumping	Pulsed or rapidly reoriented magnetic fields deform magneto‐elastic structures and trigger rapid elastic‐energy release for take‐off	Regulation of field amplitude, orientation, pulse duration, reversal rate, and release timing	Overcoming obstacles far greater than itself; instantaneous high‐energy output, reduced dependence on continuous surface contact during flight; possesses amphibious adaptability	Discontinuous; Jumping may fail due to excessive liquid adhesion; repetition rate limited by mechanical recovery and magnetic‐field switching	12, 37, 38, 39

**TABLE 2:** Representative quantitative parameters and locomotion performance of magnetic microrobots.

Motion modes	Representative robot	Dimensions	Magnetic‐field conditions	Quantitative performance	References
Translation	Cylindrical intraocular microrobot	Outer diameter: 285 μm; inner diameter: 125 μm; length: 1800 μm	20 mT; gradient: 500 mT m^−1^	Maximum displacement: 3.2 mm	16
Swimming	Helical microrobot	Body length: 135 μm	2 mT; 220 Hz	Swimming speed: approximately 3.0 mm s^−1^	26
Tumbling	Surface microroller	Nominal diameter: 7.8 μm	10 mT; 200–220 Hz	Surface locomotion speed: up to approximately 600 μm s^−1^	29
Jumping	Amphibious magnetoelastic robot	NR	Initial field: −10 mT; take‐off field: 20 mT	Jumping height: approximately 30 mm from water and 29 mm from solid ground	38

*Note*: NR: not reported in the main text. Translation was evaluated in porcine vitreous, swimming in water, and tumbling on glass submerged in PBS. Jumping results correspond to a magnetization angle of 15° and a leg angle of 45°. The reported values represent different performance metrics obtained under different experimental conditions.

### 2.1 Translational motion

Translational motion, as an extremely intuitive and extensively studied early‐stage movement mode for magnetic microrobots, relies primarily on magnetic gradient forces generated by non‐uniform magnetic fields. This differs from rotational drive mechanisms that depend on magnetic torque. Translational robots are typically composed of a highly magnetized core structure or coating. When subjected to an external gradient magnetic field, the magnetic gradient force drives them to overcome fluid resistance or surface friction, enabling movement along the gradient direction.^13^, ^14^ On solid or liquid–solid interfaces, torque‐assisted stick–slip actuation can also generate net translation by periodically overcoming static friction.^15^ The magnitude of this driving force is directly determined by the spatial gradient value of the magnetic field and the robot's total magnetic moment, granting this class of robots unique application advantages in systems capable of generating high‐gradient fields. In practice, translational motion is controlled by regulating the magnitude and direction of the magnetic‐field gradient through the currents supplied to the electromagnetic coils. When imaging feedback is available, the robot position can be used to update the target field or field‐convergence point, thereby improving trajectory‐following accuracy.

Early relevant research focused on verifying the feasibility of overcoming environmental resistance through gradient forces in microscale environments. As early as 2009, Pawashe et al. constructed a dynamic model for microrobots operating on different surfaces. They proposed generating driving torque using periodically varying pulsed magnetic fields, effectively overcoming surface static friction at the microscale through a stick–slip motion mechanism. This approach enabled two‐dimensional translation on both dry and wet surfaces.^15^ In 2013, Ullrich et al. extended the scope of translational motion research to ophthalmic surgery using an eight‐coil electromagnetic system.^16^ The cylindrical microrobot had an outer diameter of 285 μm, an inner diameter of 125 μm, and a length of 1800 μm. The OctoMag system generated magnetic fields of 10–40 mT and magnetic‐field gradients of up to 500 mT m^−1^. In porcine vitreous, the robot reached a maximum translational displacement of 3.2 mm at 20 mT and 500 mT m^−1^, whereas the maximum displacement in balanced salt solution was approximately 4 mm. These experiments demonstrated gradient‐driven translation in vitreous humor, balanced salt solution, and silicone oil under ex vivo and in vivo ocular conditions.^16^ Overcoming the complex viscoelastic resistance of the vitreous humor, this achievement laid an experimental foundation for instrument navigation in minimally invasive ophthalmic surgery. In 2024, Chen et al. designed a multilayer magnetic soft robot for sequential, on‐demand adhesion at multiple gastric ulcer sites.^17^ Magnetic gradient forces first translated the robot toward the target lesion, while interlayer magnetic attraction maintained the structural integrity of the robot during locomotion. Once the adhesive layer contacted the gastric tissue, the tissue adhesion force exceeded the interlayer magnetic coupling force. Reorientation of the external magnetic field then generated sufficient torque to separate the remaining robot from the layer adhered to the lesion, while the adhered layer remained attached to the gastric tissue, allowing the remaining robot to proceed to another target site (Figure 2a).

**FIGURE 2:**
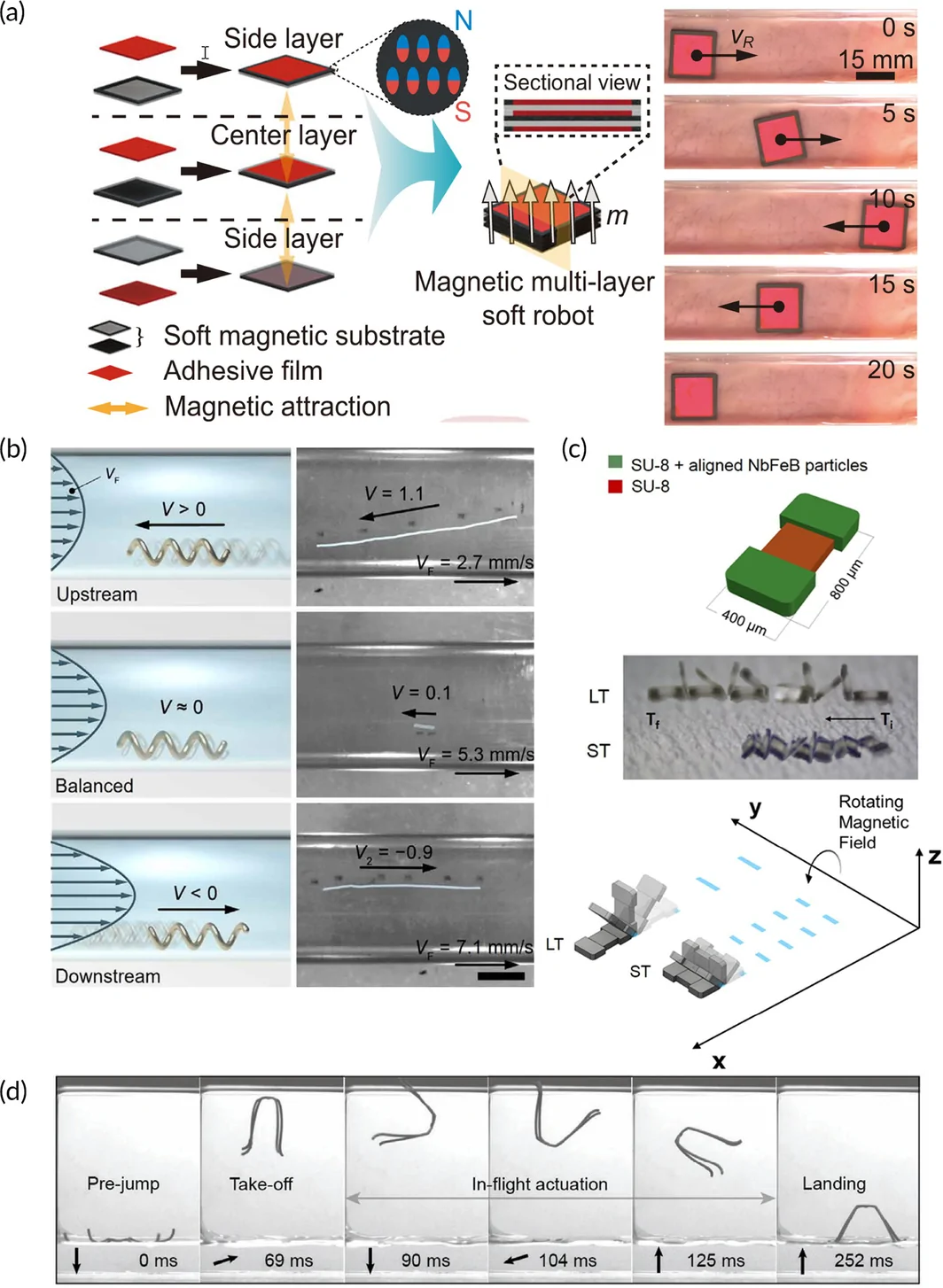
Magnetically controlled microrobot motion modes. (a) Translational motion of multilayer soft robots on gastric tissue surfaces. Reproduced under terms of the CC‐BY license.^17^ Copyright 2024, Ziheng Chen, published by Springer Nature. (b) Magnetic‐driven swimming performance of helical structure microrobots. Reproduced under terms of the CC‐BY license.^26^ Copyright 2025, Dong Wu, published by The American Association for the Advancement of Science. (c) Two motion modes of a microscale magnetic tumbling (μTUM) robot. Reproduced under terms of the CC‐BY license.^30^ Copyright 2018, Chenghao Bi, published by Multidisciplinary Digital Publishing Institute. (d) The jumping robot performs a 360° backflip on the water surface. Reproduced under terms of the CC‐BY license.^38^ Copyright 2023, Yibin Wang, published by John Wiley and Sons.

Additionally, translational motion robots have achieved breakthrough results in three key areas: interfacial collaboration, swarm control, and clinical translation. In the field of interfacial collaboration, Chen et al. developed a pair of cooperative magnetic interfacial microrobots.^18^ Using an optimized baseline feature size of approximately 8 mm, the system achieved a maximum cargo‐transport velocity of 97.5 mm s^−1^, corresponding to 12.2 body lengths per second. Under counterflow conditions, the system maintained a velocity of 8.1 body lengths per second, or approximately 65 mm s^−1^. Through optimization of magnetic field distribution and streamlined design, this system achieves non‐contact cargo capture and long‐distance translational transport. Addressing the challenge of maintaining particle swarm integrity during conventional translational motion, Cao et al. proposed an automatic control method based on rotating gradient fields for swarm control.^19^ This approach utilizes sequential excitation coils to generate a moving magnetic field convergence point, enabling rapid aggregation of microparticle clusters. By leveraging the moving convergence point, it can capture and drag particle clusters for high‐precision collective translation, effectively preventing volume loss during group translation within complex in vivo environments. In the realm of clinical translation, Landers et al. demonstrated a microrobotic platform integrated with a clinical‐grade electromagnetic navigation system.^20^ The gelatin‐based capsules had a mean diameter of 1.69 mm. The dual‐Navion system was capable of generating magnetic‐field gradients of up to 1 T m^−1^ at a field strength of 30 mT within a 20 cm × 20 cm × 20 cm workspace. In vitro bifurcation experiments evaluated navigation at mean flow velocities of 0.65–0.85 m s^−1^ and applied gradients of 0–0.45 T m^−1^. Navigation success increased with increasing magnetic‐field gradient and decreasing flow velocity, highlighting the dependence of vascular steering on magnetic actuation and hemodynamic conditions. Navigation was further demonstrated in human vascular models and intracranial vessels of live pigs.^20^

The intraocular microrobot achieved millimeter‐scale displacement under magnetic gradients of up to 500 mT m^−1^, whereas the interfacial microrobot couple reached 97.5 mm s^−1^ at the air–water interface and maintained counterflow transport at approximately 65 mm s^−1^. In the clinically oriented vascular study, the 1.69‐mm capsule was tested under flow velocities of 0.65–0.85 m s^−1^ with magnetic gradients of up to 0.45 T m^−1^, and performance was evaluated by navigation success rather than by a single propulsion speed. These results indicate that translational performance is strongly dependent on the balance between magnetic force, robot size, fluid resistance, surface interaction, and flow velocity.

Translational motion, with its intuitive control logic and efficient magnetic gradient drive mechanism, has demonstrated exceptional lesion access and payload delivery capabilities in clinical applications such as ophthalmic surgery, gastrointestinal targeted delivery, and vascular embolization. It has also achieved significant breakthroughs in multi‐robot interface collaboration and high‐precision swarm control. However, translational motion still faces physical limitations in practical applications: its driving force is highly dependent on the spatial distribution of magnetic field gradients. As distance increases, magnetic field gradients decay sharply, imposing stringent demands on the power and spatial resolution of external electromagnetic drive systems.

### 2.2 Swimming motion

In micro‐nano scale fluid environments, viscous forces dominate inertial forces. According to the scallop theorem, simple time‐symmetric reciprocating motion cannot generate net displacement.^21^ To achieve effective propulsion in such environments, magnetic microrobots often mimic the locomotion mechanisms of microorganisms like bacteria and sperm cells. They utilize rotating or oscillating magnetic fields to generate magnetic torques, driving helical structures or flexible tails to produce non‐reciprocal deformation waves, thereby enabling swimming motion. The swimming direction is determined by the rotation axis and direction of the applied magnetic field, whereas the propulsion speed is primarily regulated by the field frequency. The field amplitude determines the available magnetic torque and affects the ability of the robot to remain synchronized with the rotating field.^21^, ^22^ For flexible‐tailed swimmers, the amplitude and frequency of the oscillating field also regulate the deformation amplitude and traveling‐wave characteristics.^23^ When imaging feedback is available, the field parameters can be updated according to the robot's orientation and position to improve trajectory tracking.^24^

Helical propulsion represents a well‐established micro‐swimming approach in current research, primarily mimicking the motion of bacterial flagella. Such robots typically consist of a magnetic head and a helical tail. Under the influence of a rotating magnetic field, the helical structure converts rotational motion into axial thrust. In 2009, Zhang et al. demonstrated magnetic control of artificial bacterial flagella. A representative swimmer with a 38‐μm‐long helical tail achieved an average forward speed of 1.2 μm s^−1^ in water under a 2 mT rotating magnetic field at an angular speed of 6.3 rad s^−1^, corresponding to approximately 1 Hz.^25^ To address propulsion in blood flow, Wu et al. employed femtosecond laser‐assisted fabrication to produce hard‐magnetic helical microrobots with a body length of 135 μm (Figure 2b). In water, these robots achieved a swimming speed of approximately 3.0 mm s^−1^, equivalent to 22.6 body lengths per second, under a 2 mT rotating magnetic field at 220 Hz. In whole blood, actuation at 3 mT and 150 Hz enabled a net upstream speed of 0.5 mm s^−1^ against an average flow velocity of 5.6 mm s^−1^.^26^ To address biodegradability, Ceylan et al. developed double‐helical GelMA microswimmers using two‐photon polymerization. These microswimmers measured 20 μm in length and 6 μm in diameter and achieved an average swimming speed of 3.36 ± 0.71 μm s^−1^ in water under a 20 mT rotating magnetic field at 5 Hz. Their step‐out frequency was approximately 6 Hz under the same field amplitude, and enzyme‐mediated degradation and cargo release were demonstrated in vitro.^27^

Beyond helical structures, mimicking the undulating propulsion of sperm or eels represents another effective swimming strategy. This approach leverages distributed magnetic materials to generate traveling wave vibrations under alternating magnetic fields. Early work by Dreyfus et al. demonstrated flexible undulation achieved through chains of magnetic microspheres.^23^ Xu et al. proposed a biohybrid micromotor strategy, using magnetic streamlined‐horned caps to couple with individual spermatozoa for guided cargo delivery. This structure harnesses the natural flexibility of the sperm's flagellum as an efficient propulsion mechanism, enabling countercurrent transport of drug‐loaded sperm within simulated pulsatile blood flow environments.^28^ Hu et al. demonstrated that soft robots can flexibly switch between swimming, jumping, and tumbling based on environmental demands by integrating multimodal mechanisms, enabling adaptation to complex unstructured terrain.^12^

These studies show that magnetic‐field amplitude alone does not determine swimming performance. At the same field amplitude of 2 mT, the early artificial bacterial flagellum and the hard‐magnetic microrobot achieved water‐swimming speeds of 1.2 μm s^−1^ and approximately 3.0 mm s^−1^, respectively, but operated at markedly different frequencies and had different dimensions and magnetic configurations.^25^, ^26^ Furthermore, the biodegradable GelMA microswimmer exhibited a step‐out frequency of approximately 6 Hz at 20 mT, whereas the hard‐magnetic microrobot maintained synchronous propulsion at 220 Hz under 2 mT.^26^, ^27^ These differences highlight the need to consider robot geometry, magnetic properties, actuation frequency, and fluid conditions when comparing swimming performance.

Through biomimetic helical or oscillatory propulsion, swimming provides an effective locomotion strategy in low‐Reynolds‐number biological fluids. Although this model excels in counter‐current navigation and targeted delivery and can enhance adaptability through bio‐hybridization, its performance heavily relies on intricate 3D micro–nano structures. This results in costly fabrication processes that are difficult to mass‐produce. Simultaneously, maintaining stable propulsion efficiency and posture control in cell‐dense or high‐viscosity non‐Newtonian fluids remains a critical bottleneck awaiting breakthroughs.

### 2.3 Tumbling motion

When microrobots need to adhere to biological tissue surfaces—such as blood vessel walls or gastrointestinal tract linings—for long‐distance movement or localized operations, tumbling motion generally offers advantages over swimming motion. This locomotion primarily utilizes the magnetic torque generated by rotating magnetic fields as its driving force, converting rotational motion into forward translational traction through friction between the robot and the contact surface. In practice, tumbling motion is controlled by regulating the amplitude, frequency, rotation axis, and rotation direction of the applied magnetic field.^22^, ^29^, ^30^ The rotation plane and direction determine the tumbling trajectory, whereas the field frequency primarily controls the tumbling rate. The field amplitude must be sufficiently high to provide adequate magnetic torque and overcome the resistance associated with surface contact.^30^, ^31^

The core physical mechanism of tumbling motion follows the formula: T=m×B. When the external magnetic field rotates, the robot's magnetic moment *m* attempts to align with the magnetic field *B*, thereby generating a rotational torque. Based on the robot's geometry and magnetization axis orientation, this rotation can be categorized into two primary modes: (1) Smooth tumbling: Suitable for spherical or cylindrical robots. Alapan et al. developed multifunctional spherical Janus microrollers with diameters of approximately 3.0 and 7.8 μm for targeted recognition and light‐triggered drug release. The 7.8‐μm microrollers achieved surface locomotion speeds of up to approximately 600 μm s^−1^, corresponding to 76 body lengths per second, under a 10 mT rotating magnetic field at approximately 200–220 Hz. In whole‐blood flow at a wall shear stress of 1.2 dyn cm^−2^, microrollers with a 2‐μm‐thick Ni coating achieved an average upstream speed of 156.7 ± 36.6 μm s^−1^ at 120 Hz.^29^ Sehyuk et al. also demonstrated earlier that cylindrical soft capsules could enable semi‐implantable drug delivery through tumbling motion.^32^ (2) End‐to‐end tumbling: Suitable for elongated, dumbbell‐shaped, or irregularly shaped robots. Bi et al. designed a rectangular tumbling microrobot with a footprint of 800 μm × 400 μm that uses end‐to‐end flipping to navigate complex terrain (Figure 2c). Under a 10 mT rotating magnetic field, the robot achieved a surface locomotion speed of approximately 13.6 mm s^−1^ on a submerged substrate in water at 10 Hz. It could also traverse inclines of up to 45° on dry paper and at least 60° in water, demonstrating its ability to negotiate inclined surfaces.^30^ Building upon this approach, Niedert et al. investigated SU‐8‐ and PDMS‐based tumbling microrobots measuring 800 μm × 400 μm × 100 μm. A rotating permanent magnet provided actuation at 0.5–1.5 Hz, with an analytically estimated magnetic flux density of approximately 21.4 mT at the robot location. At 1 Hz, the lengthwise‐tumbling SU‐8 robot achieved an average speed of 2.07 ± 0.05 mm s^−1^ in the saline‐filled colon of a live mouse under ultrasound imaging. In a separate in situ test using an intact murine colon filled with 1% Tylose solution, its speed decreased to 0.21 ± 0.04 mm s^−1^ at the same frequency.^31^

The internal environment of living organisms is typically unstructured, filled with folds, mucus, and steep curved surfaces. To adapt to these environments, researchers have engineered specialized structures for tumbling robots. Wu et al. designed a wireless microrobot featuring multiple soft legs. Utilizing a magnetically driven tumbling gait, combined with mechanical interlocking and adhesive forces between the soft legs and tissue, the robot achieved stable climbing on vertical walls and even inverted surfaces within a simulated gastric model, demonstrating exceptional mobility in confined spaces.^33^ Hou et al. developed a shaftless propeller‐type magnetic millimeter robot that overcomes the limitations of single‐motion modes, enabling flexible switching between spiral swimming and surface tumbling.^34^ When encountering fluid environments, it utilizes propulsion through spirals to advance efficiently; when facing complex terrain or solid surface obstacles, programmed changes in the magnetic‐field orientation induce a transition to tumbling, enabling the robot to overcome barriers. This seamless multimodal switching between fluid and solid interfaces significantly enhances the robot's traversal capabilities in complex physiological environments.

Tumbling motion also demonstrates significant clinical potential within the gastrointestinal tract. The MagCaps capsule robot developed by Sun et al., utilizing magnetic soft valves and a dual‐layer structure design, can reach targeted lesions within the gastrointestinal tract through controlled tumbling motion, enabling precise drug delivery and fluid sampling.^35^

These studies demonstrate surface locomotion across different robot scales and actuation regimes. The 7.8‐μm microrollers reached approximately 600 μm s^−1^ at 200–220 Hz, whereas the 800‐μm‐long tumbling robots achieved approximately 13.6 mm s^−1^ on a submerged substrate at 10 Hz and 2.07 ± 0.05 mm s^−1^ in a saline‐filled murine colon at 1 Hz.^29^, ^30^, ^31^ These results should be compared in the context of robot dimensions, magnetic‐field conditions, substrate properties, and fluid environments. The substantially lower speed observed in the Tylose‐filled colon further illustrates the sensitivity of tumbling performance to the surrounding medium and surface‐contact conditions.^31^

Tumbling motion leverages the synergy between magnetic torque and friction to achieve efficient adhesion and flexible navigation on biological tissue surfaces. While this approach excels in obstacle‐climbing and targeted therapy, it remains highly sensitive to interfacial friction conditions: it tends to slip and fail on super‐lubricated mucus surfaces (e.g., thick mucus layers), while encountering excessive starting resistance in highly adhesive environments. Maintaining sustained adhesion and motion stability across complex, irregular topographies remains a primary challenge limiting its application.

### 2.4 Jumping motion

When encountering complex, unstructured topographies within biological organisms—such as gastrointestinal folds or vascular walls—traditional translational or tumbling motions often struggle to traverse due to surface friction or geometric constraints. Inspired by organisms like springtails and nematodes that utilize an energy accumulation‐instantaneous release mechanism to achieve explosive movement in nature, jumping motion has emerged as a key strategy for microrobots to overcome macroscopic obstacles.^36^ Unlike continuous swimming or crawling motions, jumping movements primarily rely on the nonlinear deformation properties of magnetoelastic composite materials. They achieve transient high‐energy output through the conversion between magnetic potential energy and elastic potential energy.^37^ The core driving principle is generally based on the mutual antagonism between magnetic torque and elastic restoring force. When a specific external magnetic field is applied, the robot undergoes severe bending or curling deformation due to the magnetic torque, accumulating elastic potential energy. Subsequently, by rapidly reversing the magnetic field direction or removing the magnetic field, the structure induces instability or violent rebound. This causes the robot to instantaneously release stored energy and impact the substrate, generating a reaction force that propels it into a mid‐air leap.^12^ Jumping is therefore actuated by time‐varying magnetic fields that deform the magnetoelastic body and trigger rapid energy release. The take‐off performance is mainly regulated by the strength and orientation of the initial and take‐off magnetic fields, whereas the forward trajectory can be controlled by changing the pitch angle of the take‐off field.^38^

Hu et al. proposed a soft millirobot with multimodal locomotion capabilities. The representative robot measured 3.7 mm × 1.5 mm × 0.185 mm and consisted of an elastomer matrix embedded with neodymium‐iron‐boron particles. Its programmed single‐wavelength harmonic magnetization profile enabled complex deformation under external magnetic fields. Directional jumping was demonstrated using a time‐varying magnetic‐field sequence with a magnitude of 16.5 mT, coupling elastic deformation with rigid‐body rotation to generate an impulsive impact against the substrate.^12^ To improve locomotion in complex environments involving both liquid and solid interfaces, amphibious jumping robots have been investigated. Wang et al. developed an amphibious miniature soft jumping robot comprising four superhydrophobic magnetoelastic legs (Figure 2d). The robot had a body mass of 33 mg. For a robot with a magnetization angle of 15° and a leg angle of 45°, an initial magnetic field of −10 mT followed by a take‐off magnetic field of 20 mT produced jumping heights of approximately 30 mm from the water surface and 29 mm from solid ground.^38^ Additionally, bistable structures have been used to enhance take‐off velocity and enable rapid elastic energy release in magnetic jumping robots.^39^

These studies demonstrate different jumping mechanisms under magnetic fields of comparable magnitude. Hu et al. demonstrated directional jumping of a 3.7‐mm‐long soft robot using a 16.5 mT magnetic‐field sequence that coupled elastic deformation with rigid‐body rotation.^12^ Wang et al. achieved jumping heights of approximately 30 mm from water and 29 mm from solid ground using an initial field of −10 mT followed by a 20 mT take‐off field.^38^ These results highlight that magnetic‐field amplitude alone is insufficient to characterize jumping performance. Robot dimensions, magnetization configuration, magnetic‐field sequence, and take‐off interface should also be considered.

Jumping motion, based on a bio‐inspired energy storage‐instantaneous release mechanism, effectively addresses the challenge of traditional continuous motion being easily obstructed in unstructured terrains such as tissue folds. This mode has achieved amphibious adaptability from dry surfaces to wet confined spaces, significantly expanding the operational boundaries of microrobots. However, the trajectory of jumping motion exhibits discrete characteristics, making micrometer‐level continuous precise positioning difficult. Moreover, how to efficiently overcome fluid viscosity and surface adhesion forces in wet environments to maintain stable energy release remains a major challenge limiting its further application.

## 3 MATERIAL SELECTION

The core challenge in developing material systems for magnetic microrobots in medical applications lies in achieving an optimal balance between strong magnetic responsiveness and superior biocompatibility. Ideal materials not only possess sufficient magnetic moment to generate driving force but also maintain chemical stability within complex physiological environments, thereby avoiding immune responses or cytotoxicity.

### 3.1 Magnetic functional materials

Magnetic functional materials serve as the core physical medium enabling energy conversion and drive control in microrobots. Based on differences in hysteresis loop characteristics and magnetization state stability, these materials are typically categorized into soft magnetic materials and hard magnetic materials. The distinctions between these two types in magnetic properties, drive mechanisms, and biocompatibility collectively form the physical foundation for micro‐robots to achieve multimodal motion, environmental sensing, and integrated diagnosis and treatment.

#### 3.1.1 Soft magnetic materials

In the material systems for magnetic microrobots, soft magnetic materials have become fundamental functional materials for constructing in‐body interventional devices due to their unique hysteresis characteristics and excellent biocompatibility. Soft magnetic materials (such as Fe_3_O_4_, Fe, NiFe, etc.) exhibit physical characteristics including high magnetic permeability, low coercivity, and low remanence.^8^, ^40^, ^41^ This endows them with exceptional responsiveness to external magnetic fields: they rapidly magnetize to generate strong induced magnetic moments when a magnetic field is applied, and their macroscopic magnetism vanishes almost instantly upon field removal (Figure 3a). This magnetic switch property effectively prevents nonspecific aggregation of the microrobots due to magnetic dipole interactions in the absence of a magnetic field, thereby significantly reducing the risk of thrombosis and blockage during in vivo applications.^2^, ^42^

**FIGURE 3:**
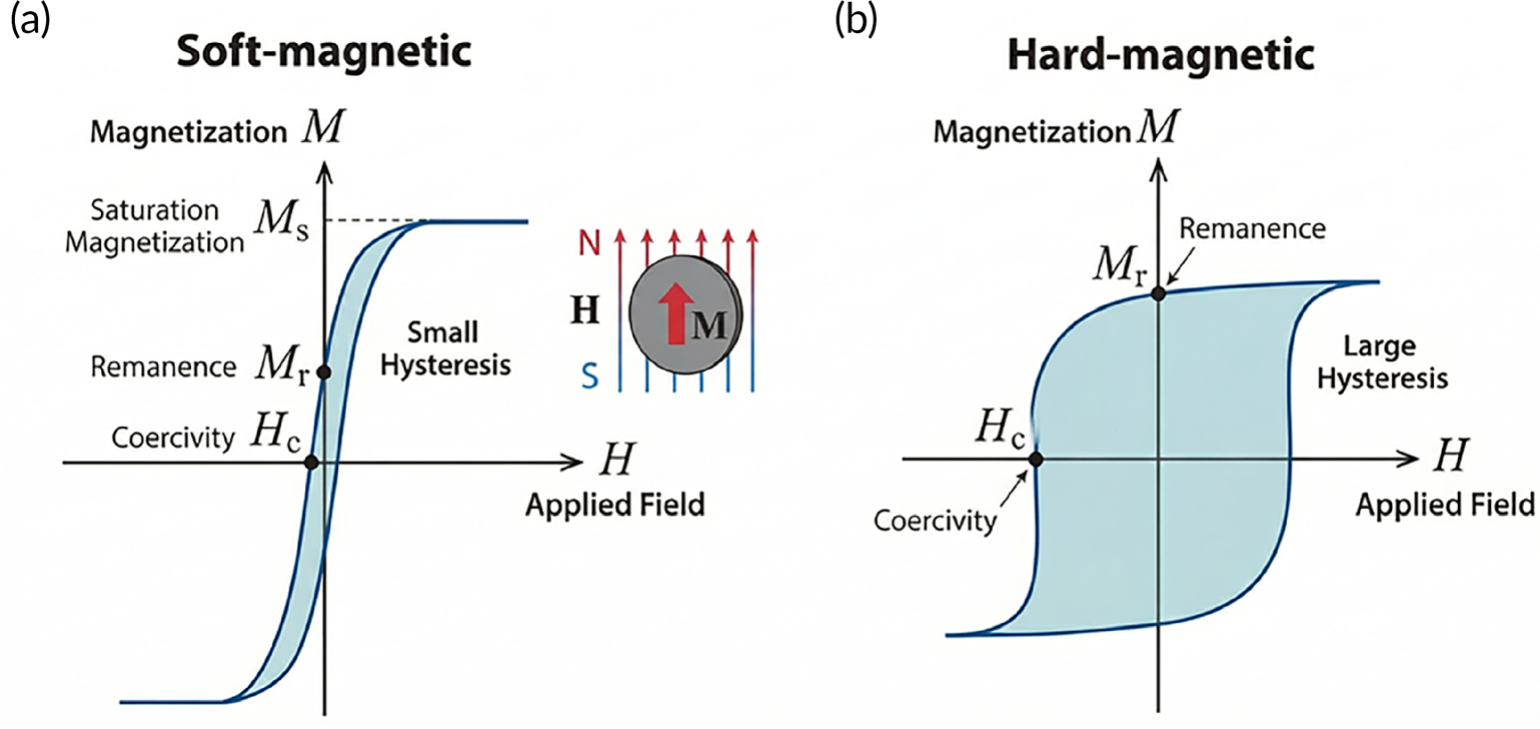
Hysteresis loop diagram of magnetic functional materials. (a) Hysteresis loop of soft magnetic materials. Reproduced with permission.^40^ Copyright 2022, American Chemical Society. (b) Hysteresis loop of hard magnetic materials. Reproduced with permission.^40^ Copyright 2022, American Chemical Society.

The macroscopic magnetism of soft magnetic materials originates from the evolution of their microscopic magnetic domain structure. According to classical ferromagnetic domain theory, these materials exhibit low magnetic crystal anisotropy constants and minimal lattice defects. This allows domain walls to readily migrate and coalesce with oppositely oriented domains under an external magnetic field, resulting in extremely low coercivity.^43^ When the size of soft magnetic particles decreases below the single‐domain critical size (typically tens of nanometers), the thermal energy in the environment becomes sufficient to overcome the magnetic anisotropy barrier within the particles. This causes the magnetic moments to randomly flip in the absence of an external field, resulting in a macroscopically zero magnetic moment.^37^, ^44^ This superparamagnetic property enables nanoscale soft magnetic particles to achieve hysteresis‐free on‐demand magnetization, where the relationship between magnetization and external field follows the Langevin function, ensuring precise controllability of magnetic driving forces.^8^, ^37^

The propulsion of soft magnetic microrobots primarily relies on induced magnetization. Within a gradient magnetic field, the magnetic traction force they experience is proportional to the product of the magnetic field gradient ($F=\left(m\cdot \nabla \right)B$), making them commonly used for point‐to‐point translational operations.^42^

In a uniform rotating magnetic field, soft magnetic materials typically struggle to generate rotational torque directly like hard magnetic materials because they cannot maintain a fixed internal magnetization direction. However, this issue can be effectively addressed by designing shape‐anisotropic, non‐spherical geometries (such as elongated chain‐like or ellipsoidal structures). When the external magnetic field direction is misaligned with the microrobot's geometric long axis, the robot experiences a magnetic torque that rotates its long axis parallel to the magnetic field direction to minimize the energy of the residual demagnetization field within its body.^21^, ^45^ Although soft magnetic robots exhibit slightly lower drive efficiency than hard magnetic robots at extremely high frequencies, soft magnetic helical robots based on shape anisotropy still demonstrate superior propulsion performance within the low‐frequency range without requiring complex magnetization programming steps.^25^

#### 3.1.2 Hard magnetic materials

In the material systems of magnetic microrobots, hard magnetic materials exhibit extremely high coercivity and remanence (Figure 3b). This physical property enables hard magnetic materials, once magnetized, to “memorize” specific magnetic moment directions and intensities like permanent magnets. Even when the external field is removed or undergoes drastic changes in direction, their internal magnetic domain structures remain highly stable without flipping.^40^, ^41^ This physical property endows hard magnetic microrobots with two core advantages: first, an extremely high magnetic moment density, enabling them to generate driving forces several times stronger than soft magnetic materials, effectively overcoming the high viscous resistance of biological fluids; second, unique magnetization programming capability, allowing for the pre‐programming of complex and discrete magnetic moment distributions within the robot body—the physical foundation for achieving high‐dimensional motion control in soft robots. Furthermore, hard magnetic materials do not require magnetization–demagnetization cycles, enabling faster dynamic responses under high‐frequency rotating magnetic fields. Recent studies demonstrate that hard‐magnetic helical microrobots can achieve a net upstream speed of 0.5 mm s^−1^ against a blood flow velocity of 5.6 mm s^−1^.^26^

The superiority of hard magnetic materials stems from their high coercivity and strong magnetic anisotropy. Taking typical NdFeB as an example, its internal magnetic domains exhibit exceptional stability after magnetization. The direction and magnitude of the magnetic dipole moment *m* remain virtually unchanged under perturbations from an external low‐frequency driving magnetic field *B* (i.e., *m* is a constant vector). Consequently, the magnetic torque acting on the microrobot strictly follows the vector cross‐product law: T=m×B. This implies that the driving torque depends on the magnitude and direction of its embedded magnetic moment and the applied magnetic field, independent of the robot's geometric shape.^45^ This characteristic greatly liberates the structural design freedom of microrobots, enabling even spherical, cubic, or highly symmetrical structures to generate substantial rotational torque by setting specific magnetization directions.^22^

By dispersing hard magnetic particles within a flexible polymer matrix (such as PDMS or Ecoflex) and applying a strong magnetic field with a specific orientation during fabrication, magnetic particles in different regions can be aligned along a predetermined direction. This enables the construction of non‐uniform, discrete, or even continuously varying magnetic pole distributions within a continuous medium.^45^

When a weaker driving magnetic field (far below the material's coercivity) is applied, magnetic moments in different regions of the robot attempt to align simultaneously with the external field. Due to differing directions of magnetic moments at various points, the resulting local magnetic moments counteract each other, generating a complex internal stress field within the soft body. This internal stress drives the robot to undergo continuous deformations such as bending, folding, and twisting, rather than simple rigid body rotation.

Although hard magnetic materials like NdFeB possess absolute advantages in performance, their clinical translation faces a major obstacle: biocompatibility. NdFeB contains the heavy metal neodymium, and iron‐based alloys are highly susceptible to oxidation and corrosion in physiological environments (oxygen‐rich, saline solutions). This not only leads to magnetic property degradation but also releases metal ions that may induce cytotoxicity.^42^, ^46^, ^47^ Therefore, constructing a dense and biologically inert surface encapsulation layer is an essential step in the design of hard magnetic microrobots. Current solutions include: utilizing chemical vapor deposition technology to prepare a dense conformal Parylene C coating on the NdFeB surface. Recent research confirms that this pinhole‐free polymer film effectively blocks ion leaching, demonstrating superior corrosion resistance and in vivo biocompatibility compared to traditional gold plating layers^48^, ^49^; alternatively, bioactive surfaces can be constructed using titanium‐based nanostructured titanates, which simultaneously block heavy metal toxicity while significantly enhancing the adhesion and transport capabilities of microrobots for cellular cargo.^50^ Additionally, encapsulating magnetic particles within a biodegradable hydrogel matrix represents an emerging strategy. This approach enables the controlled separation and recovery of magnetic components upon completion of targeted delivery tasks through stimuli such as near‐infrared light.^51^

### 3.2 Matrix materials

As the primary structural framework for magnetic microrobots, matrix materials serve not only as physical carriers for magnetic functional units and various payloads (such as drugs and cells), but also determine the robots' biocompatibility, motion flexibility, and environmental responsiveness. This section focuses on hydrogels, elastomers, biodegradable polymers, shape‐memory polymers, liquid metals, and other functional matrices used in magnetic microrobots (Table 3).

**TABLE 3:** Magnetically controlled microrobot matrix materials.

Matrix category	Representative material	Key characteristics	Limitations	Application advantages	References
Natural polymer hydrogels	Alginate	Bivalent cation cross‐linking mechanism; extremely high water content; tunable pore size; low immunogenicity	Poor mechanical properties; lack of enzymatic degradation within the body; unstable ionic cross‐linking	Suitable for the rapid preparation of microspheres using microfluidic technology; protects drugs from gastric acid; gentle conditions that do not damage cells	54, 55, 56
	Chitosan	Polycationic properties; antibacterial and hemostatic; soluble in acid; precipitates or gels in neutral environments	Poor solubility; brittle when dry, low strength and prone to swelling when wet; prone to causing blood clots	Suitable for microrobots used in infection control or wound healing; pH‐sensitive drug‐releasing carriers	57, 58
	Gelatin/GelMA	Thermal reversibility; bioactive sites: rich in RGD peptides (promoting cell adhesion) and MMP sites (enzymatic cleavage)	Thermal instability; poor mechanical properties; excessive degradation rate; severe deformation due to water absorption	GelMA‐based bio‐inks for 3D printing and photolithography; drug delivery for cartilage repair; cell delivery and tissue‐engineering applications	53, 59
	Silk Fibroin	High tensile strength and toughness; controllable degradation; excellent optical transparency	Residual silk protein can cause severe inflammation, and the delamination process is cumbersome; it is extremely difficult to dissolve	Form a high‐strength scaffold; suitable for long‐term implantation	60
Synthetic responsive hydrogels	PNIPAM	Hydrophilic swelling occurs below the LCST; hydrophobic collapse and shrinkage occur above the LCST	Slow response; Poor biodegradability; monomers are toxic and require rigorous purification	Develop an integrated robot; enable passive triggering	61, 62
	pH‐sensitive hydrogels (PAA/PMAA)	Rich in carboxyl groups; structurally stable and more resistant to enzymatic hydrolysis than natural materials	Slow response time; non‐biodegradable; susceptible to masking by salt ion concentrations in the body, resulting in a failure to swell	Improve the survival rate of orally administered macromolecular drugs; when combined with magnetic properties, they can be made into open‐and‐close capsules	54, 61
Elastomers	PDMS and Ecoflex	Low modulus; High stretchability; reversible deformation; compatibility with magnetic particles	Generally non‐biodegradable or poorly resorbable	Programmable deformation and locomotion	12, 45, 63, 64, 65
Biodegradable polymers	PLGA	Adjustable degradation rate; good biocompatibility; high mechanical strength	Poor water solubility; intrinsic degradation; acidic degradation products	The top choice for sustained‐release systems; suitable for 4D printing	67
	PCL	Excellent flexibility and elongation; extremely slow degradation rate; easy to process	Low mechanical strength; highly hydrophobic; degrades too slowly	Commonly used for braces requiring long‐term mechanical support	53
Shape‐memory polymers	Thermally responsive SMPs and magnetic SMP composites	Temporary/permanent shape programming, stimulus‐responsive shape recovery	Require thermal or other external stimulation	4D‐printed shape morphing, deployment and remote reconfiguration	68, 69
Other functional matrices	Magnetic viscoelastic fluids	Magnetorheological effect; viscoelastic matrix support; non‐contact control	Temperature sensitivity; susceptibility to oxidation; matrix compatibility	Ability to navigate extremely confined spaces; reduced mechanical damage to surrounding tissues	70
	Liquid metals	High electrical and thermal conductivity; low toxicity; low melting point and good flowability	Extremely high surface tension; risk of leakage; difficult to control adhesion	Deformation and merging using combined magnetic fields	71
	Edible/bio‐degradable substrates	Fully biodegradable; provide gas barrier properties, helping to prevent food oxidation	Poor mechanical properties; extremely high water sensitivity; poor thermal stability	Gastrointestinal diagnostics and treatment with zero risk of residue	72
	Gelatin‐based biodegradable biogels	High resilience; fracture toughness and room‐temperature/laser‐assisted healing	Magnetic integration and microscale fabrication require further validation	Sustainable soft robots and biodegradable electronic platforms	73

#### 3.2.1 Hydrogels

Hydrogels are a class of three‐dimensional polymer networks with high water content (up to 90% or more). Their physical properties—such as low elastic modulus, surface wettability, and high diffusivity—closely resemble those of human soft tissues, making them one of the most biocompatible matrix materials.^52^, ^53^ In magnetic microrobots, the unique network structure of hydrogels makes them ideal platforms for drug delivery and cell culture, enabling loading and release at scales ranging from molecules to cells.

##### Natural polymer hydrogels

Natural polymer hydrogels, derived from biomacromolecules such as gelatin, alginates, chitosan, and silk fibroin, are widely utilized due to their excellent biocompatibility and inherent biodegradability.

**Alginate**: Alginate is a natural polysaccharide whose unique divalent cation cross‐linking mechanism makes it highly suitable for rapid fabrication of microsphere or capsule‐shaped robots via microfluidic technology. This egg carton structure endows the matrix with excellent mechanical strength and pH sensitivity, enabling it to remain compact in gastric acid (pH <3) to protect the payload while swelling and disintegrating in the neutral intestinal environment (pH >7), thereby achieving targeted drug release.^54^ Aybar Tural et al. developed a magnetic alginate microrobot based on droplet microfluidic technology (Figure 4a). This study leveraged an internal gelation mechanism to achieve high‐throughput and highly monodisperse fabrication of the microrobots, effectively addressing the issues of low yield and difficult size control in traditional methods.^55^ Li et al. further proposed a multifunctional Fe‐alginate microgel robot that efficiently encapsulates single cells. By leveraging the permeability of the alginate matrix, it maintains cellular viability and proliferative capacity, offering a novel delivery vehicle for cell therapy.^56^

**FIGURE 4:**
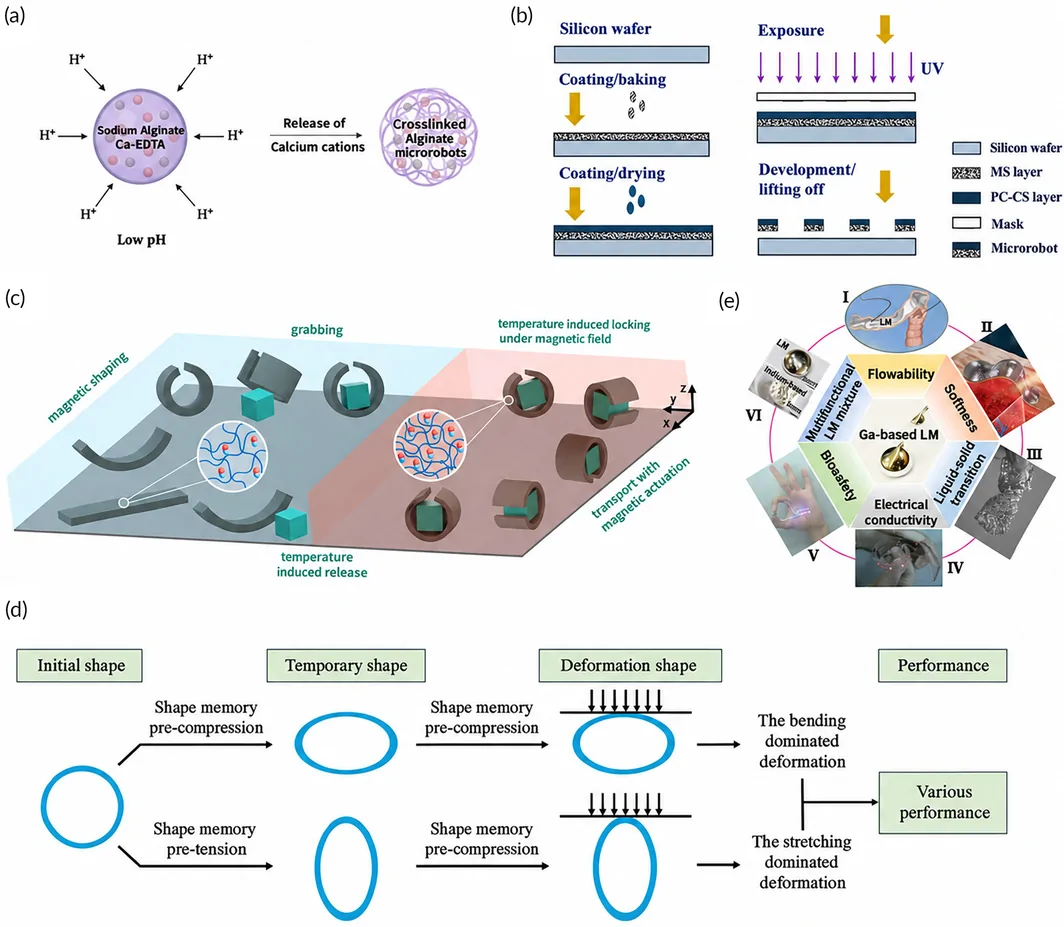
Matrix materials for magnetic microrobots. (a) Highly monodisperse alginate magnetic microspheres. Reproduced with permission.^55^ Copyright 2025, Elsevier. (b) Fabrication of non‐chiral planar microrobots based on chitosan. Reproduced under terms of the CC‐BY license.^58^ Copyright 2022, Haoying Wang, published by MDPI. (c) Response behavior of the PNIPAM‐based THANOS robot to thermal and magnetic stimuli. Reproduced under terms of the CC‐BY license.^62^ Copyright 2024, Clio Siebenmorgen, published by Royal Society of Chemistry. (d) Schematic diagram illustrating changes in the structure, shape, and properties of TSMP. Reproduced under terms of the CC‐BY license.^68^ Copyright 2025, Jian Li, published by Taylor & Francis. (e) Physical properties of gallium‐based liquid metals. Reproduced with permission.^71^ Copyright 2018, John Wiley and Sons.

**Chitosan**: Chitosan is the only naturally occurring polysaccharide with a positive charge, enabling it to form strong adhesion through electrostatic interactions with negatively charged glycoproteins on biological mucosal surfaces, thereby prolonging the residence time of robots in the gastrointestinal tract or respiratory tract. To overcome the low mechanical strength of pure chitosan, Veloso et al. developed a chitosan/alginate polyelectrolyte composite magnetic gel. Through a pH‐triggered self‐assembly process, this composite matrix not only achieved uniform dispersion of magnetic nanoparticles but also attained a storage modulus as high as 10 kPa. This effectively enabled sustained release of anticancer drugs (such as 5‐fluorouracil) and magnetic hyperthermia therapy.^57^ Wang et al. fabricated a dual‐layer microrobot composed of chitosan and magnetic particles using photolithography (Figure 4b). By leveraging the pH‐responsive properties of the chitosan layer, they achieved intelligent drug loading and controlled release, effectively addressing the challenge that traditional photolithography‐based microrobots face in drug delivery due to the biological inertness of their materials.^58^

**Gelatin**: Gelatin preserves the cell adhesion sites (RGD sequence) and matrix metalloproteinase (MMP) degradation sites of collagen, making it an ideal material for constructing tissue engineering robots. GelMA modified by methacrylation combines the biological activity of natural gelatin with the photopolymerization capability of synthetic polymers.^53^ Wei et al. developed a biodegradable magnetic microrobot based on alginate/GelMA microcapsules for the delivery of chondroitin sulfate, enabling targeted cartilage tissue repair under magnetic‐field guidance.^59^

**Silk fibroin**: Silk fibroin possesses a unique *β*‐sheet structure, endowing it with mechanical strength far exceeding that of conventional hydrogels and a tunable degradation cycle (ranging from weeks to years). Maia et al. addressed the limitations of natural silk fibroin—their susceptibility to enzymatic degradation and lack of smart responsiveness—by proposing a silk fibroin‐polymer/inorganic hybrid strategy. This approach not only significantly enhanced the material's mechanical strength and degradation resistance but also endowed it with intelligent responsiveness to pH or temperature changes. Consequently, it effectively enabled precise, controlled drug release and facilitated bone tissue repair.^60^

##### Synthetic responsive hydrogels

To endow microrobots with enhanced environmental adaptability, synthetic hydrogels (such as PEGDA, PVA, and PNIPAM) and smart responsive materials have been incorporated into matrix design. These materials feature precisely engineered chemical structures and are capable of undergoing phase transitions or changes in modulus in response to physical or chemical stimuli (such as temperature, light, or magnetic fields).^61^ PNIPAM is the most classic thermosensitive hydrogel; its volume phase‐change properties near its lower critical solution temperature (LCST; approximately 32°C) have been widely utilized to construct micro‐grippers with dual “magneto‐thermal” responsiveness. The minimalist PNIPAM/magnetic particle composite robot (Figure 4c) designed by Siebenmorgen et al. can be navigated using a magnetic field and triggers matrix contraction via localized thermal stimulation to grasp microparticles, demonstrating its application value in single‐cell manipulation.^62^ In response to changes in the pH gradient of the gastrointestinal tract, pH‐sensitive hydrogels based on polyacrylic acid (PAA) or polymethacrylic acid (PMAA) have been used to develop oral drug delivery robots. These robots remain contracted in gastric acid to protect the drug and expand upon entering the intestine to release it, thereby effectively improving the bioavailability of macromolecular drugs.^54^

#### 3.2.2 Elastomers

Elastomers are soft polymeric materials capable of large, reversible deformations, making them attractive matrices for flexible magnetic microrobots.^63^ Silicone elastomers, such as polydimethylsiloxane (PDMS) and Ecoflex, are commonly used because of their compliance, stretchability, and ability to incorporate magnetic particles.^45^, ^64^ By embedding hard‐magnetic particles in an elastomer matrix and programming their magnetization directions, external magnetic fields can induce deformation and locomotion in soft robots. For example, Hu et al. developed a multimodal soft microrobot comprising an elastomer matrix embedded with neodymium–iron–boron particles. Its programmed magnetic response enabled reversible deformation and transitions among different locomotion modes.^12^ However, conventional PDMS‐based silicone elastomers are generally considered non‐biodegradable or non‐resorbable in biomedical applications,^65^ which may limit their use when post‐treatment degradation or resorption is required. Thus, elastomer selection should consider elastic recovery, mechanical durability, compatibility with magnetic particles, biocompatibility, and the intended residence time of the microrobot.^45^, ^64^, ^66^

#### 3.2.3 Biodegradable polymers and shape‐memory polymers

To prevent foreign body reactions caused by the prolonged retention of microrobots in the body, polymer matrices with controllable degradation profiles have become a hot topic of research. Poly (lactic‐co‐glycolic acid) (PLGA) and polycaprolactone (PCL) are currently the most widely studied biodegradable matrices.^53^ Among these, PLGA, due to its excellent biocompatibility and tunable degradation rate, has been shown to effectively reduce sudden drug release and enable sustained drug delivery.^67^ Building on this, 4D printing technology has been introduced to endow materials with dynamic response properties, thereby enabling them to adapt to complex physiological environments. Li et al. noted that thermally triggered shape‐memory polymers (SMPs) can achieve morphological reconfiguration and stiffness modulation through thermal activation (Figure 4d).^68^ Kortman et al. reviewed strategies for programming the orientation and magnetization of magnetic fillers within polymer matrices, including shape‐memory polymer composites, to achieve field‐induced shape morphing.^69^

#### 3.2.4 Other functional matrices

In an effort to overcome the physical limitations of traditional solid polymers, emerging matrix materials with extreme deformability and unique biological properties have become a hot topic of research. Sun et al. proposed a magnetic viscoelastic fluid (Slime) based on a polyvinyl alcohol–boric acid network, which leverages its non‐Newtonian fluid properties to achieve extreme passive deformation within confined spaces and self‐healing capabilities following fracture.^70^ To address the integration needs of flexible electronics, Wang et al. developed a liquid–metal composite substrate (Figure 4e), which, thanks to its excellent conductivity and damage resistance, endows soft robots with integrated diagnostic and sensing capabilities such as electromagnetic sensing and Joule heating actuation.^71^ In addition, in pursuit of optimal biosafety, Wu et al. developed a fully digestible, edible bio‐substrate that allows the robot to be broken down by the body's enzymatic system and absorbed as nutrients after completing gastrointestinal intervention tasks, thereby fundamentally eliminating the risk of the device remaining in the body for an extended period.^72^ To enable the sustainable manufacturing of soft robots and address the issue of electronic waste, Baumgartner et al. developed a fully biodegradable gelatin‐based biogel.^73^ This biogel exhibits mechanical resilience comparable to that of common synthetic silicone elastomers, as well as high fracture toughness, and it can be rapidly healed through laser‐assisted local melting.

## 4 MANUFACTURING METHODS

The manufacturing process for microrobots is a critical step in translating design concepts into tangible functionality. Given that their dimensions span a wide range from the micro‐ and nano‐scale to the millimeter scale, and involve the heterogeneous integration of soft and hard magnetic materials, hydrogels, and active biomaterials, no single manufacturing technology can meet all requirements. Currently, the field has developed a diversified manufacturing system centered on additive manufacturing (3D/4D printing), template‐assisted deposition, microfluidic technology, and self‐assembly techniques^41^ (Table 4).

**TABLE 4:** Comparison of manufacturing processes for magnetically controlled microrobots.

Manufacturing methods	Advantages	Limitations	References
Additive manufacturing			
Two‐photon polymerization (TPP)	Facilitates complex 3D geometries with sub‐micron precision; capable of producing pure metal structures through high‐temperature sintering	Inherently low throughput, hindering mass production; laser scattering caused by magnetic fillers may compromise structural fidelity	74
Direct ink writing (DIW)	Suitable for the fabrication of soft robots; In situ magnetization programming enables voxel‐level control of magnetic axis orientation	Limited spatial resolution, posing challenges for micro‐scale feature definition	76
Template‐assisted electrochemical deposition			
Porous membrane template	Easy to prepare multi‐segment structures; surface is suitable for multifunctional modification	Confined by template channel geometry (mostly cylindrical); incapable of generating complex 3D topologies	77, 78
3D photolithography template method	High material purity; excellent magnetic properties; high structural fidelity	Multi‐step procedural complexity involving sacrificial layers and casting	79
Microfluidic technology			
Droplet microfluidics	Good mono‐dispersity (uniform particle size); suitable for biological/cell encapsulation	Restricted primarily to spherical or simple ellipsoidal geometries	82, 83
Continuous‐flow lithography	Produce complex 2D cross‐sections and continuously fabricate particles with complex two‐dimensional cross‐sections	Geometric constraints limit outputs to 2D extruded profiles	84
Assembly technology			
Magnetic self‐assembly	Possess exceptional abilities to transform, split, and merge; highly adaptable to its environment	Limited structural rigidity; requires high‐precision spatiotemporal control of magnetic fields	85, 86
Modular voxel assembly	Facilitates the integration of heterogeneous materials with distinct physical properties; transcends the material limitations of single‐process fabrication	Requires precise voxel positioning, jig‐assisted assembly, and bonding	87

### 4.1 Additive manufacturing

Additive manufacturing has become the mainstream choice for microrobot fabrication due to its ability to construct three‐dimensional geometries of any complexity. Depending on the scale, the primary methods are two‐photon polymerization (TPP) and direct ink writing (DIW).

TPP technology utilizes femtosecond lasers to induce localized two‐photon absorption in photosensitive resins, enabling sub‐micron‐scale ultra‐high‐resolution fabrication. However, directly incorporating magnetic nanoparticles into photosensitive resins can cause laser scattering and compromise structural fidelity. To address this limitation, Li et al. proposed a hybrid fabrication strategy combining two‐photon polymerization with high‐temperature sintering (Figure 5a). They first printed a nickel‐containing precursor scaffold, then removed the organic binder and reduced the nickel metal through high‐temperature sintering, successfully fabricating high‐performance pure‐nickel microrobots.^74^ In addition, Wang et al. proposed a bio‐inspired fish‐shaped microrobot based on micro‐nano 4D printing technology (Figure 5b). By spatially programming magnetically responsive composites and pH‐sensitive hydrogels, this study not only achieved flexible magnetically controlled swimming but also enabled the tail joint to deform in response to environmental pH, thereby intelligently regulating drug release.^75^

**FIGURE 5:**
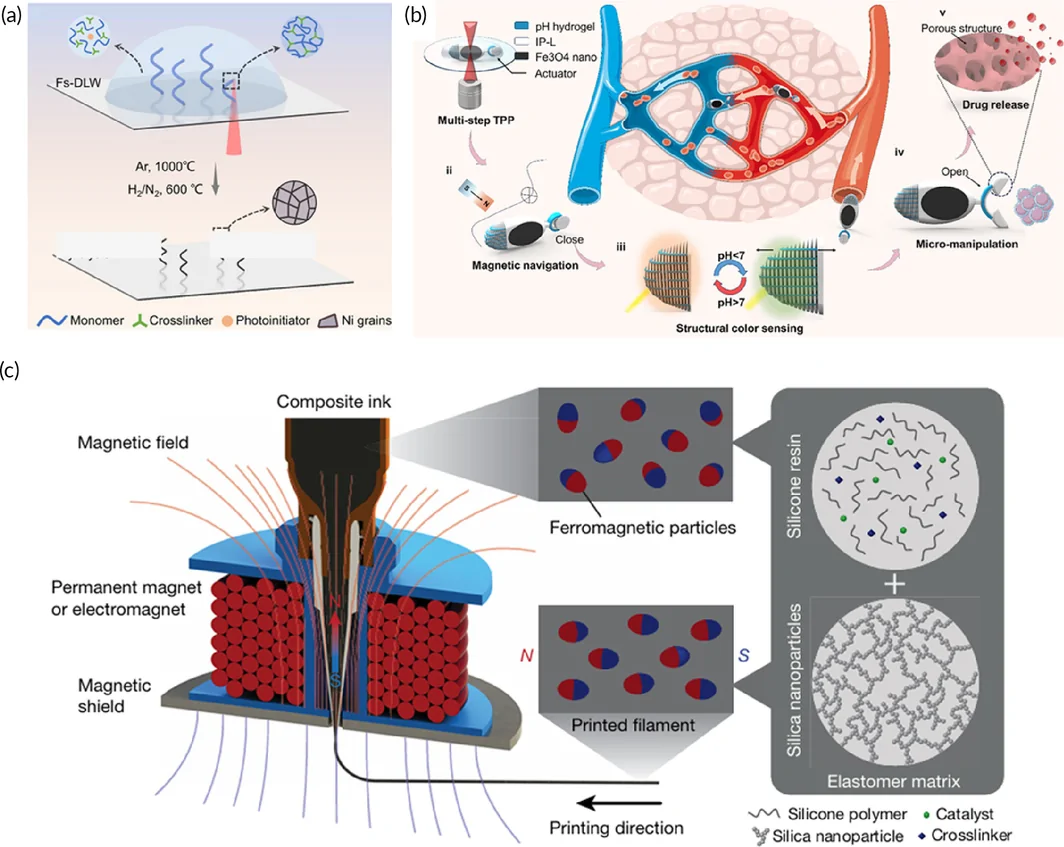
Additive manufacturing strategies for magnetic microrobots. (a) Fabrication of nickel‐based microspirals by Fs‐DLW, followed by high‐temperature sintering and reduction to produce Ni‐MAR. Reproduced with permission.^74^ Copyright 2024, Royal Society of Chemistry. (b) Schematic diagram of a 4D‐printed microrobot with multimodal synergy. Reproduced with permission.^75^ Copyright 2025, John Wiley and Sons. (c) Schematic diagram of the DIW process and the principles of ferromagnetic domain programming. Reproduced with permission.^76^ Copyright 2018, Springer Nature.

For millimeter‐scale soft robots, DIW technology enables the direct extrusion of viscoelastic ink containing magnetic particles. Kim et al. pioneered the application of a dynamic magnetic field around the DIW nozzle (Figure 5c) to adjust the orientation of the magnetic particles in real time before the ink solidifies.^76^ This in situ magnetic programming technique enables each point of the printed filament to have an independent magnetic axis, thereby endowing soft robots with the ability to undergo complex, continuous deformation.

### 4.2 Template‐assisted electrochemical deposition

Template‐assisted electrochemical deposition, as a “bottom‐up” additive manufacturing strategy, utilizes micro‐ and nano‐scale polymers or sacrificial layers as templates. Through electrochemical reactions, materials are deposited into the interior of the template or onto its surface, thereby enabling the high‐fidelity fabrication of complex three‐dimensional structures. The porous membrane template method utilizes the nanopores of anodized aluminum oxide (AAO) or polycarbonate (PC) filter membranes as templates to fabricate magnetic nanowires via electrochemical deposition. In particular, by periodically altering the composition of the electrolyte, “barcode” nanowires composed of different metals (e.g., Au‐Ni‐Au) can be fabricated (Figure 6a). This approach not only enables magnetic actuation but also provides the capability for multifunctional surface modification, making it commonly used for intracellular detection and sensing.^77^, ^78^

**FIGURE 6:**
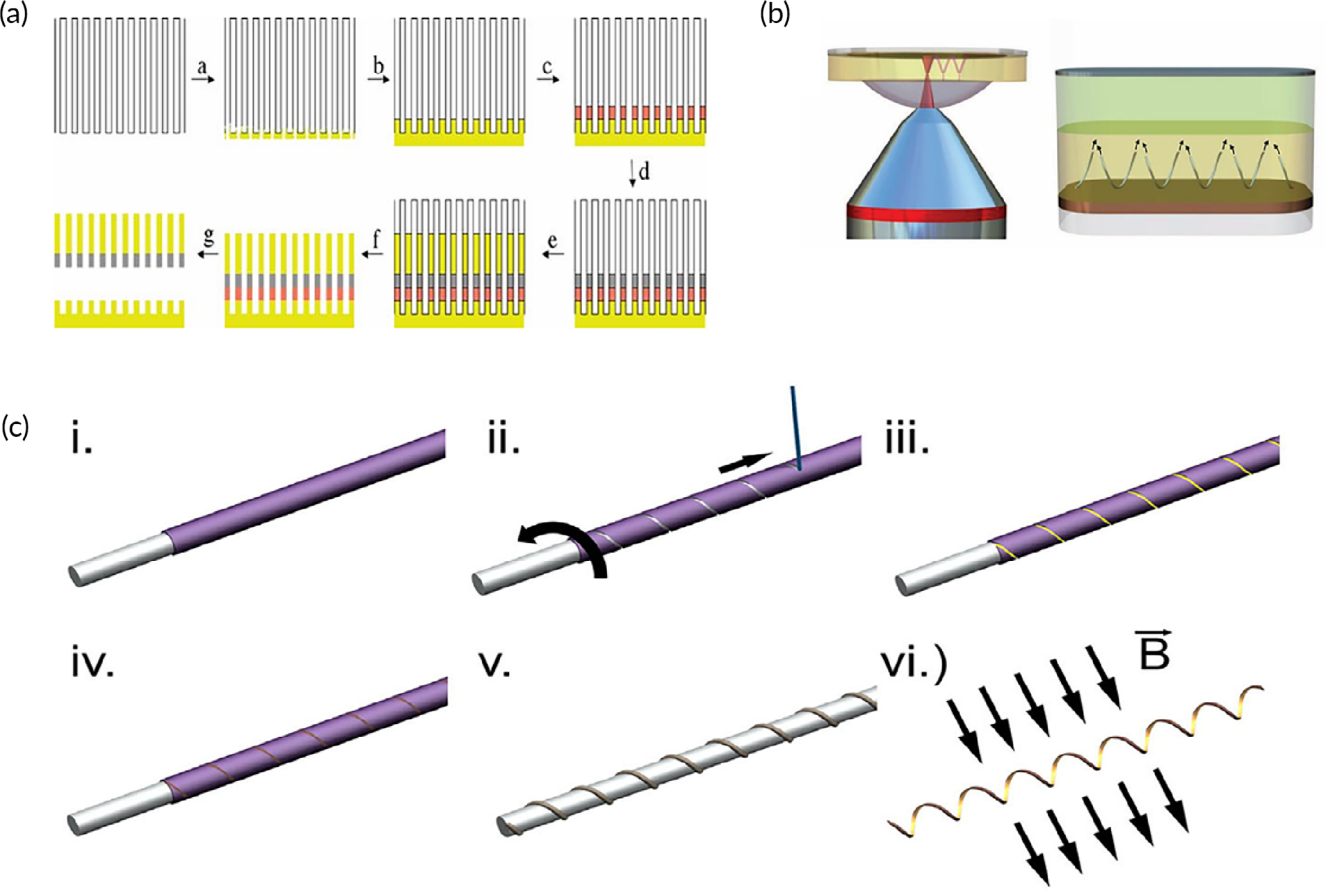
Template‐assisted electrochemical deposition methods. (a) Schematic diagram of nano‐barcode synthesis. Reproduced with permission.^78^ Copyright 2005, Royal Society of Chemistry. (b) Metal components manufactured using 3D‐assisted electroplating. Reproduced with permission.^79^ Copyright 2019, John Wiley and Sons. (c) Procedures for the fabrication of artificial bacterial flagella (ABFs). Reproduced with permission.^80^ Copyright 2018, John Wiley and Sons.

To enhance the biosafety and propulsion performance of microrobots, Alcântara et al. successfully fabricated pure iron microrobots using an electrochemical deposition process based on 3D photolithography templates (Figure 6b). Compared to nickel or cobalt, which have potential toxicity, iron exhibits a higher saturation magnetization and excellent biocompatibility.^79^ In addition, this process also supports the integration of semi‐hard magnetic materials. Chatzipirpiridis et al. used laser ablation of a polymer coating to fabricate a core mold, which they combined with an electroforming process to produce cobalt‐platinum (CoPt) alloy microhelices (Figure 6c), thereby endowing the robot with more stable magnetic properties.^80^

### 4.3 Microfluidic technology

Microfluidic technology uses micron‐scale channels to precisely manipulate fluids and has become a key process for manufacturing hydrogel‐based micro‐ and nanorobots that is efficient, low‐cost, and capable of extremely high throughput.^81^ Unlike point‐by‐point two‐photon polymerization, microfluidic fabrication typically relies on the principle of multiphase flow, which involves the formation of discrete droplets or laminar flow within microchannels using immiscible fluids (such as an oil phase and a water phase), combined with photopolymerization or ionic cross‐linking to achieve rapid curing and shaping of microstructures.

Microfluidic droplet generation is currently one of the most widely used strategies for fabricating microrobots. A review by Shang et al. noted that by precisely controlling the flow rate ratio between the dispersed phase and the continuous phase, as well as the channel geometry (such as T‐junctions or flow‐focusing structures), it is possible to generate microdroplets with high size uniformity (mono‐modality) at frequencies as high as several thousand hertz.^82^ A significant advantage of this technology lies in its ability to create complex internal topologies. Shah et al. developed a design emulsion technique based on glass capillaries, utilizing a W/O/W (water‐in‐oil‐in‐water) double emulsion template to fabricate microcapsules with a core–shell structure in a single step (Figure 7a). This structure is particularly well‐suited for the construction of drug‐carrying microrobots, as the shell provides protection while the core is used to encapsulate highly active biopharmaceuticals or cells.^83^ Recent research advances have further validated this potential: Wang et al. successfully developed single‐cell microgel robots using microfluidic technology (Figure 7b). By encapsulating individual stem cells together with magnetic nanoparticles within alginate microgels, these microrobots not only possess magnetically driven targeting capabilities but can also be tracked in vivo via MRI imaging, enabling the precise delivery and visual guidance of cell therapies.^56^

**FIGURE 7:**
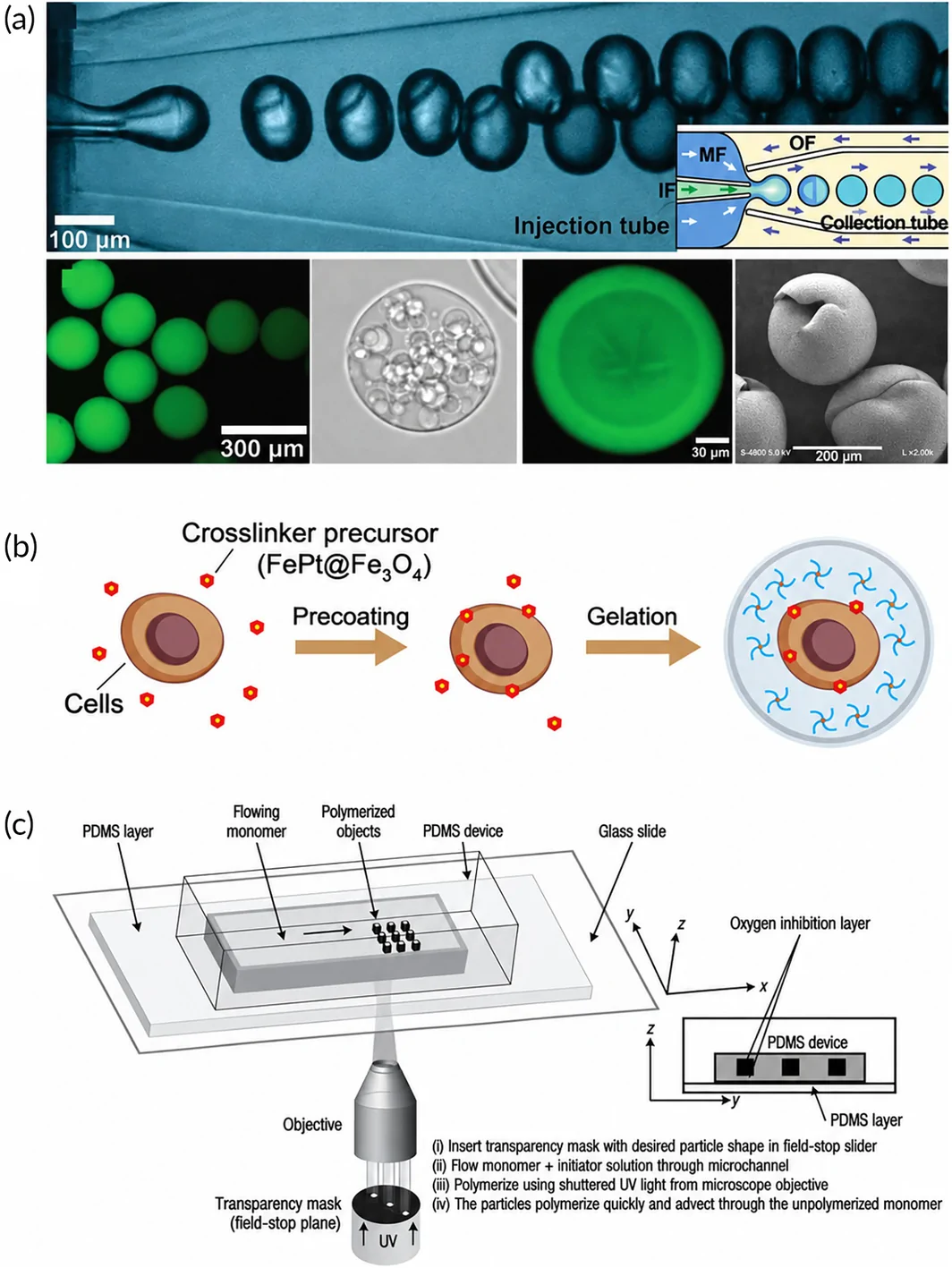
Microfluidic approaches for monodisperse particle fabrication, cell microencapsulation, and high‐throughput microparticle synthesis. (a) Synthesis of monodisperse particles in microfluidic devices. Reproduced under terms of the CC‐BY license.^83^ Copyright 2008, Rhutesh K. Shah, published by Elsevier. (b) The various steps in the cell microencapsulation process. Reproduced under terms of the CC‐BY license.^56^ Copyright 2025, Xiuyu Wang, published by Chinese Chemical Society. (c) Schematic diagram of an experimental setup for high‐throughput synthesis of micrometer‐sized particles using continuous‐flow lithography. Reproduced with permission.^84^ Copyright 2006, Springer Nature.

Although droplet microfluidics excels at fabricating spherical or capsule‐shaped structures, it has limitations when it comes to manufacturing non‐spherical microrobots. To overcome this challenge, Dendukuri et al. proposed a continuous‐flow photolithography technique (Figure 7c). This method combines microfluidics with projection lithography, using a microscope objective to project UV light with specific shapes (such as triangles, stripes, or even encoded patterns) onto a flowing stream of photosensitive prepolymer.^84^ This in situ UV exposure and photopolymerization curing method within microchannels enables the continuous fabrication of polymer particles with complex two‐dimensional cross‐sections, offering a wide range of morphological options for microrobots and effectively enhancing their adaptability to complex fluid environments.

### 4.4 Assembly strategies

When building reconfigurable systems, assembly techniques offer unique advantages. Magnetic self‐assembly harnesses magnetic dipole interactions to drive millions of magnetic nanoparticles to spontaneously assemble into chain‐like, vortex‐like, or ribbon‐like clusters within a fluid. These liquid robots exhibit exceptional adaptability to their environment and can split and merge in response to changes in channel width.^85^ Ussia et al. demonstrated the use of rotating magnetic fields to control the assembly of functionalized magnetic microspheres into traps for the efficient capture of microplastics and bacteria in water.^86^ In addition to magnetic self‐assembly, modular voxel assembly can address the limitations of single‐material functionality. Zhang et al. proposed a voxel‐based assembly strategy in which cubic modules with different magnetization directions or functions are prefabricated and then assembled into complex three‐dimensional machines using jig‐assisted positioning and bonding (Figure 8). This method enables the fabrication of heterogeneous multi‐material systems that are difficult to achieve with conventional printing, such as composite robots that simultaneously possess hard magnetic drive capabilities and soft drug‐delivery functions.^87^

**FIGURE 8:**
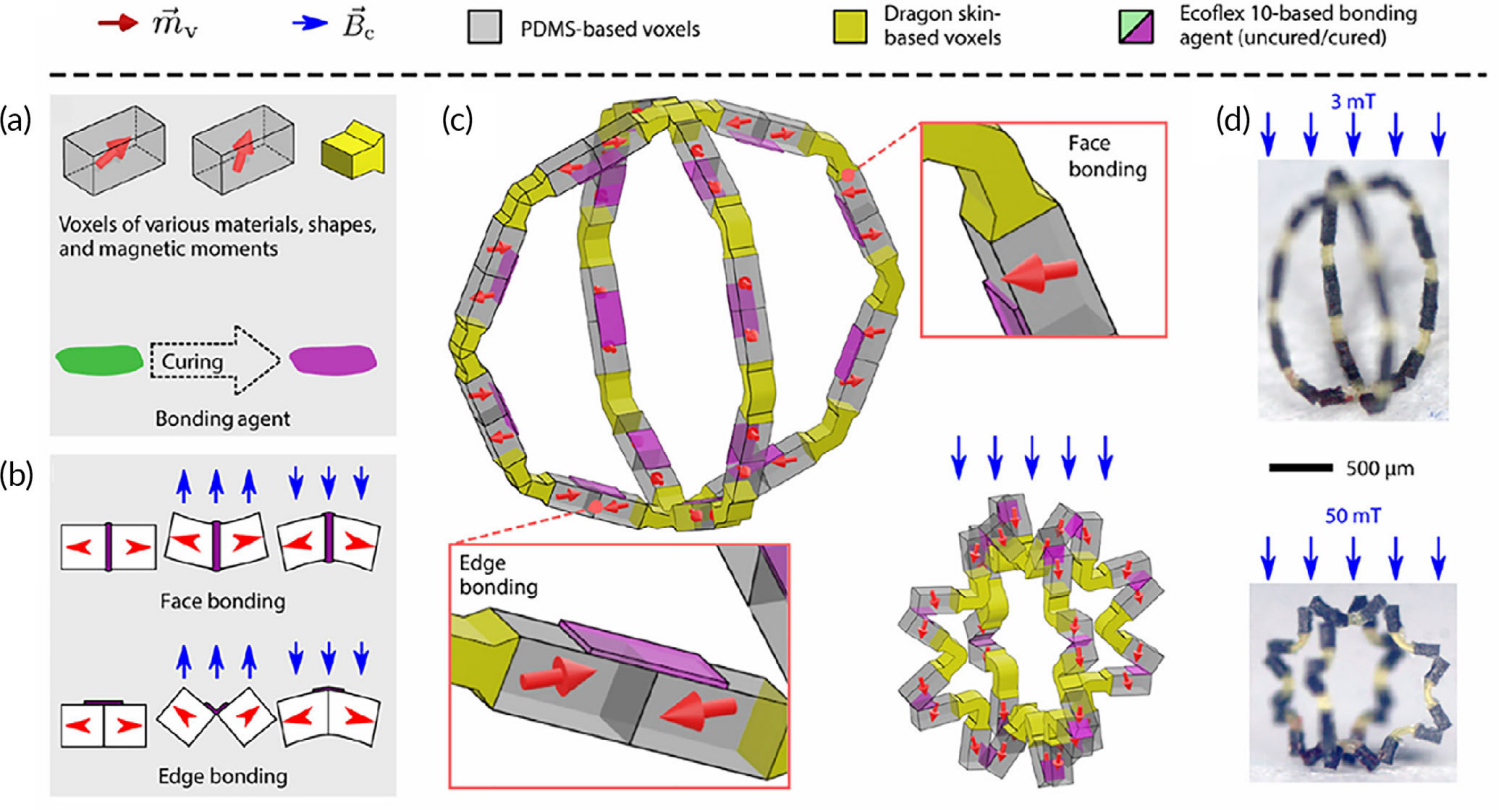
A jig‐assisted heterogeneous integration method for 3D manufacturing of small‐scale magnetic soft machines. (a) Voxels are bonded together to construct soft machines. (b) Adhesives connect adjacent voxels in two different ways: face bonding and edge bonding. (c) By assembling a number of heterogeneous voxels at specified 3D positions and orientations, a soft machine with two interconnected rings is fabricated. (d) The soft machine demonstrated 3D mechanical metamaterial properties. Reproduced with permission.^87^ Copyright 2021, The American Association for the Advancement of Science.

## 5 MEDICAL APPLICATIONS AND CLINICAL VALIDATION

With the growing popularity of precision medicine, minimally invasive and targeted approaches have become major trends in the development of modern medicine. Magnetically controlled microrobots, with their untethered propulsion, sub‐millimeter size, and exceptional maneuverability, have overcome the limitations of traditional catheters and endoscopes in terms of physical dimensions and operational freedom, providing a novel interventional approach for the diagnosis and treatment of deep‐seated lesions. This chapter will systematically discuss the current status of clinical validation of magnetically controlled microrobots, focusing on three major applications: vascular intervention, gastrointestinal diagnosis and treatment, and cancer therapy (Table 5).

**TABLE 5:** Clinical applications of magnetically controlled microrobots.

Application scenarios	Specific application	Representative robot types	Clinical advantages	Key challenges	References
Vascular intervention	Retrograde navigation	Hard‐magnetic helical microrobots; surface microrollers; on‐wall‐rotating millirobots; magnetic mobile microrobots; magnetic microrobotic swarms	Bypasses the geometric and mechanical constraints of conventional catheters in reaching distal microvasculature	High hemodynamic shear forces; rapid pulsatile flow; complex vascular tortuosity and branching	26, 29, 92, 93, 94
	Targeted thrombolysis	Nanoparticle‐shelled magnetic microbubbles; rotating helical magnetic swimmers	Reduces the risk of systemic bleeding and improves thrombolytic efficacy	Low drug penetration; thrombosis and sclerosis	95, 96
	Vascular embolism	Magnetic soft microfiberbots; thermoresponsive magnetic nanorobot collectives; magnetic microrobotic swarms	More effectively fills irregular lesions to prevent aneurysm rupture	The aneurysm is irregular in shape and difficult to fill completely	90, 97, 98
Gastrointestinal diagnosis and treatment	Targeted delivery	Endoscopy‐assisted biohybrid soft microrobots; capsule‐type magnetic microrobots	Enable rapid intervention for deep‐seated lesions (such as in the colon); non‐invasive oral administration of macromolecular drugs	Prolonged anatomical transit; vigorous peristaltic dynamics; extreme pH fluctuations causing premature degradation	91, 99
	Biopsy procedure	Magnetic capsule endoscopes carrying thermoresponsive microgrippers; magnetically actuated soft capsule endoscopes; magnetic robotic biopsy endoscopes; capsule‐like puncture biopsy robots	Overcoming the limitations of superficial sampling to enable deep tissue biopsy and thermal hemostasis	Difficulty in locating the target during passive movement	100, 101, 102, 103
	Repair and regeneration	Multifunctional magnetic soft robots carrying therapeutic patches	Protects against acidic environments and provides customized repair for large, irregular wounds	Corrosion of the wound surface in a strongly acidic environment and a high inflammatory response	104
Cancer treatment	Physical therapy	TriMag microrobots; biodegradable MXene‐based magnetic microrobots	Zero or minimal medication; prevention of drug resistance; real‐time tracking	Difficult to achieve deep penetration; limited efficacy with monotherapy	105, 106
	Chemotherapy/immunotherapy	Magneto‐aerotactic bacterial carriers; magnetic hydrogel microrobots; biohybrid blood‐hydrogel fiber robots; immune‐cell‐based biohybrid microrobots	Increases drug concentration within the tumor; reduces systemic side effects; activates the immune response	Low drug permeability; high systemic toxicity; hypoxic microenvironment	107, 108, 109, 110
	Mechanical destruction	Magnetic‐vortex microdiscs; magnetic nanomotor swarms	Physical disruption of tumor cells and intracellular structures	Solid tumor stromal barrier; drug resistance	111, 112
Other scenarios	Ophthalmic surgery	Slippery magnetic micropropeller swarms	Achieves precise delivery of drugs to specific regions of the retina	Viscosity of the vitreous body impedes diffusion	116
	Tissue engineering	Porous magnetic cell‐carrying microrobots; stem‐cell‐based medical microrobots; neural‐cell‐carrying magnetic microrobots	Improves stem cell engraftment and restores neural signal transmission	Low cell retention rate; disrupted neural connections	117, 118, 119
	Anti‐infective	Catalytic antimicrobial robots based on iron oxide nanoparticle aggregates	Effectively removes stubborn biofilms and prevents secondary infections	Biofilms are highly resistant to antibiotics	121

Beyond navigation and targeted delivery, the therapeutic efficacy of magnetic microrobots also depends on how the payload is released after reaching the target site. Representative mechanisms include pH‐responsive release based on matrix swelling, dissolution, or structural changes^54^, ^88^; enzyme‐/degradation‐mediated release through the cleavage and breakdown of biodegradable matrices^27^; thermoresponsive or magnetic‐hyperthermia‐mediated release through gel–sol transition, enhanced molecular diffusion, or phase‐change‐material melting^57^, ^89^, ^90^; and magnetically triggered mechanical release through the controlled opening of a capsule chamber or valve.^91^ As summarized in Table 6, endogenous triggers such as pH and enzymes provide physiological or pathological specificity but are susceptible to biological variability, whereas externally applied thermal or magnetic stimuli offer improved temporal control but require careful regulation of heating and structural actuation.

**TABLE 6:** Representative drug‐release strategies in magnetic microrobots and related magnetic delivery systems.

Drug release strategy	Triggering mechanism	Advantages	Limitations	References
pH‐responsive release	Matrix swelling, dissolution, or structural change under specific pH conditions	Suitable for site‐specific gastrointestinal release	Sensitive to local pH, ionic strength, and physiological variability	54, 88
Enzyme‐/degradation‐mediated release	Enzymatic cleavage or hydrolytic degradation induces matrix swelling and network breakdown, thereby releasing the encapsulated cargo	Biodegradable and potentially responsive to pathological enzyme levels; Enables complete cargo release	Release depends on local enzyme concentration and degradation kinetics; Premature or variable release may occur	27
Thermoresponsive and magnetic‐hyperthermia‐mediated release	Thermal or magnetic heating induces matrix transition or phase‐change‐material melting, or enhances polymer relaxation and drug diffusion	Remote and externally controllable release; compatible with magnetic targeting and release‐rate modulation	Requires careful temperature control to avoid thermal damage	57, 89, 90
Magnetically triggered mechanical release	Magnetic force or torque opens a capsule chamber, valve, or layered structure	Remote, on‐demand, and spatially localized release	Requires precise magnetic control and reliable structural switching	91

### 5.1 Interventional vascular therapy

Blood vessels serve as an ideal pathway for microrobots to perform long‐distance transport and targeted interventions. Compared to traditional catheter technologies, magnetically controlled microrobots offer advantages such as tether‐free operation, miniature size, and agile movement, enabling them to penetrate peripheral micro‐vessels that are inaccessible to microcatheters. However, the intravascular environment is highly dynamic and complex. The impact of pulsatile blood flow, interference from dynamic obstacles such as red blood cells, and the unstructured nature of vascular branches pose significant challenges to the microrobots' ability to navigate against the flow, their targeted positioning mechanisms, and their functional execution modules.

#### 5.1.1 Retrograde navigation in a blood flow environment

To navigate through arteries or veins, microrobots must first overcome the resistance of blood flow. For micro‐ and nanoscale swimmers, blood exhibits significant viscosity, and flow velocities often far exceed the robots' self‐propulsion speeds. To address this challenge, Wu et al. used femtosecond laser two‐photon polymerization to fabricate hard magnetic helical microrobots with extremely high coercivity, successfully achieving stable counter‐current swimming in simulated blood flow at sub‐centimeter‐per‐second speeds.^26^ Alapan et al. utilized the low‐velocity boundary layer near the vessel wall to significantly reduce fluid resistance through a surface tumbling mechanism.^29^ Yang et al. proposed a wall‐hugging rotation strategy, designed a streamlined millimeter‐scale robot, and utilized the no‐slip boundary conditions at the vessel wall to convert fluid drag into a frictional driving force, thereby achieving stable counter‐current motion in a highly dynamic environment.^92^ Wu et al. conducted a systematic study of the size and morphological effects of microrobots. By optimizing the parameters of the rotating magnetic field, they achieved precise navigation of the microrobots, switching between upstream and downstream directions within complex microfluidic chips and the blood vessels of live zebrafish (Figure 9a).^93^

**FIGURE 9:**
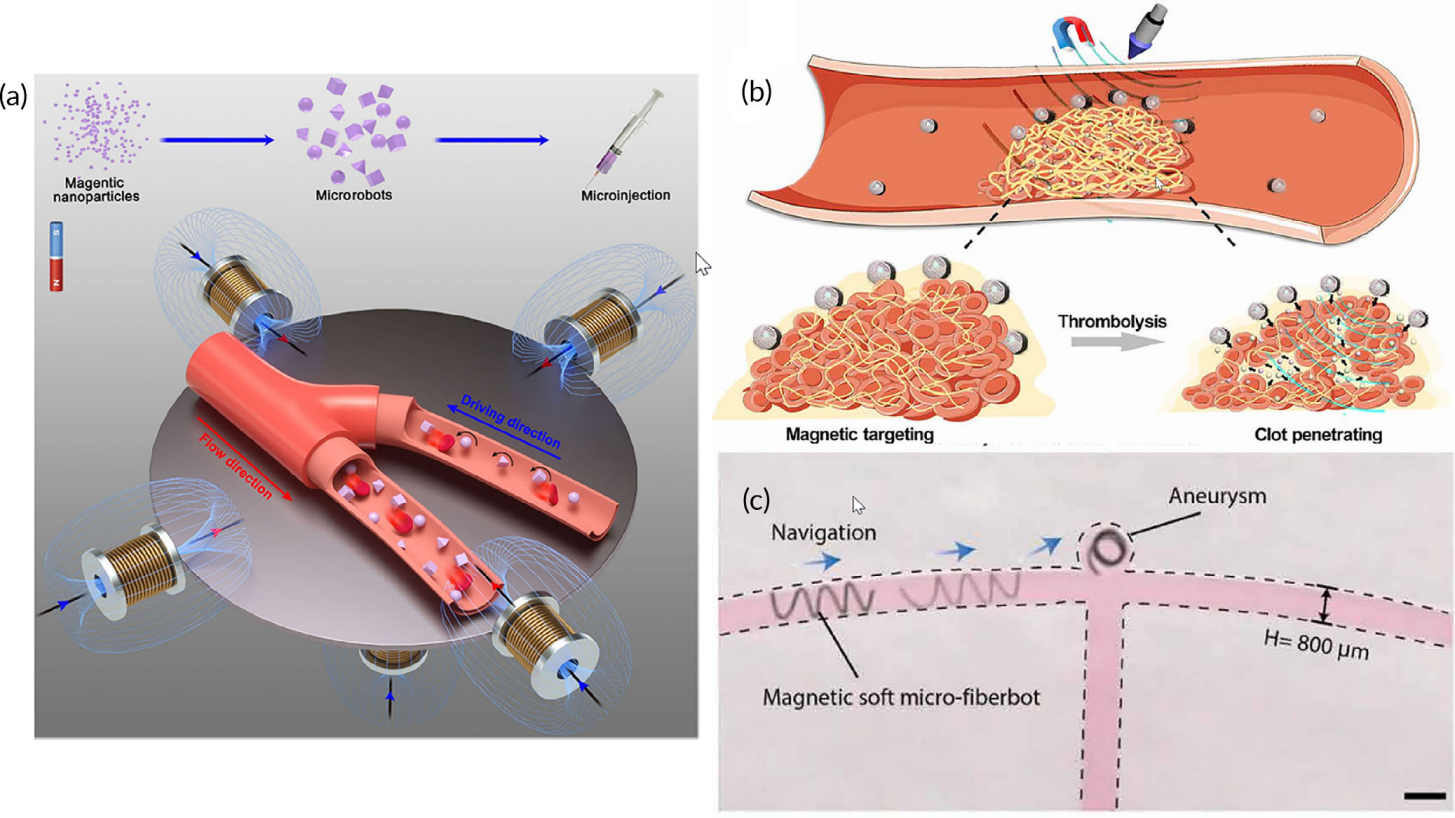
Representative magnetic microrobot strategies for vascular navigation, targeted thrombolysis, and aneurysm embolization. (a) Schematic diagram of the navigation of a magnetically controlled microrobot in blood vessels. Reproduced under terms of the CC‐BY license.^93^ Copyright 2022, Zehao Wu, published by John Wiley and Sons. (b) Illustration of targeted delivery and controlled release for thrombolytic. Reproduced with permission.^95^ Copyright 2020, The American Association for the Advancement of Science. (c) Demonstration of aneurysm coil embolization using a magnetic soft microfiber robot. Reproduced with permission.^97^ Copyright 2024, The American Association for the Advancement of Science.

However, in complex pulsating flow environments, individual robots often suffer from insufficient driving force, while magnetic swarms face the risk of structural collapse. To address this, Law et al. developed a propulsion strategy based on an oscillating magnetic field to tackle the challenge of structural collapse in magnetic nanoparticle swarms within fluid flow. By adjusting the magnetic field strength and pitch angle to maintain the swarm's structural integrity, they successfully achieved counter‐current navigation and targeted recovery of the swarm in simulated blood flow.^94^

#### 5.1.2 Targeted thrombolysis and thrombectomy

Thromboembolism is a major cause of stroke and myocardial infarction. Traditional pharmacological thrombolysis (such as tPA injection) carries a risk of systemic bleeding, whereas microrobots can achieve targeted, highly efficient combined physical–chemical thrombolysis. Wang et al. designed a swarm of microbubble robots loaded with tPA (Figure 9b). Under the influence of a rotating magnetic field, these microbubbles can aggregate and “grind” the thrombus surface, while ultrasound is used to trigger microbubble rupture to enhance drug penetration into the interior of the thrombus, thereby increasing thrombolytic efficiency several‐fold.^95^ For old, hardened blood clots, medication alone is often of limited effectiveness. Leclerc et al. have developed a helical magnetic microrobot with a specialized tip design that can physically penetrate calcified thrombus tissue like a miniature drill.^96^

#### 5.1.3 Treatment of vascular embolism and aneurysms

Vascular embolization is widely used to treat aneurysms, arteriovenous malformations, or to cut off the blood supply to tumors. Compared to traditional metal coils, soft microrobots can more tightly fill irregular lesion spaces. Liu et al. reported a magnetic soft microfiber robot (Figure 9c). Under magnetic field guidance, this fiber robot can form a dense physical barrier within the aneurysm sac and induce thrombus formation, thereby supporting aneurysm embolization.^97^

To further enhance the stability and safety of embolization, Wang et al. proposed a thermally responsive magnetic nanorobot swarm. Upon reaching the target blood vessel, the cluster uses focused magnetic hyperthermia to melt the PCM, releasing thrombin to rapidly induce thrombus formation and complete the aneurysm embolization.^90^ This system exemplifies a magnetic‐hyperthermia‐mediated phase‐change release strategy. In a study on microvascular embolization, Law et al. demonstrated the ability to dynamically construct a blocking wall at vascular bifurcations using clusters of micrometer‐sized magnetic particles, offering a new approach to temporary vascular occlusion.^98^

### 5.2 Gastrointestinal diagnosis and treatment

As the body's largest immune organ and the primary channel for nutrient absorption, the gastrointestinal tract presents significant challenges for the deployment and operation of microrobots due to its winding and extensive anatomical structure, vigorous physiological peristalsis, and a chemical environment characterized by drastic fluctuations in pH levels. Traditional capsule endoscopes are limited by their passive, flow‐dependent movement, making it difficult to achieve targeted drug delivery or tissue biopsy. In contrast, magnetically controlled microrobots, leveraging the advantages of active navigation and multifunctional integration, have opened up a new technological pathway for the precise diagnosis and treatment of gastrointestinal diseases.

#### 5.2.1 Targeted delivery and non‐invasive diagnosis and treatment

In addressing the delivery challenges posed by the complex physiological environment of the gastrointestinal tract, magnetic microrobots have demonstrated the potential for precise diagnosis and treatment that surpasses that of traditional oral formulations. Faced with the challenge of low transport efficiency over long distances within the entire gastrointestinal tract, relying solely on the autonomous movement of microrobots often makes it difficult to rapidly reach deep lesions (such as the colon). To address this, Wang et al. proposed an endoscope‐assisted navigation strategy that uses clinical endoscopes to rapidly transport a swarm of microrobots to the target area for release, thereby enabling rapid intervention and imaging in the deep intestine.^99^ To further protect sensitive drugs or cellular carriers, Lee et al. designed a capsule‐shaped microrobot capable of encapsulating drugs or neural cells within a sealed chamber for undamaged transport; upon reaching the target site, the capsule is opened via magnetic field control to release its payload.^91^ This design exemplifies a magnetically triggered mechanical release strategy.

#### 5.2.2 Biopsy and minimally invasive surgery

In addition to diagnostic observation, performing biopsies on suspicious tissues within the digestive tract or conducting minimally invasive surgeries are key tasks in interventional therapy. Sehyuk et al. proposed a micro‐gripper strategy based on a release‐retrieval mechanism, successfully obtaining multiple mucosal samples from the gastric surface using a temperature‐sensitive self‐folding gripper.^100^ To address the need for deep tissue sampling, Son et al. developed a magnetically controlled soft capsule that uses a magnetic field to deform the capsule body and eject a fine needle, thereby overcoming the limitations of superficial sampling and enabling fine‐needle aspiration biopsy of the submucosal layer.^101^ In addition, Hoang et al. designed a biopsy capsule robot incorporating a helical drive mechanism (Figure 10a), which uses an external rotating magnetic field to drive an internal screw to extend a cylindrical biopsy blade for cutting and retract it to store the sample. The robot successfully performed precise tissue biopsies in an ex vivo porcine gastric model.^102^ Xu et al. designed a puncture capsule with integrated thermal hemostasis, which effectively reduced the risk of postoperative bleeding while enabling deep tissue sampling.^103^

**FIGURE 10:**
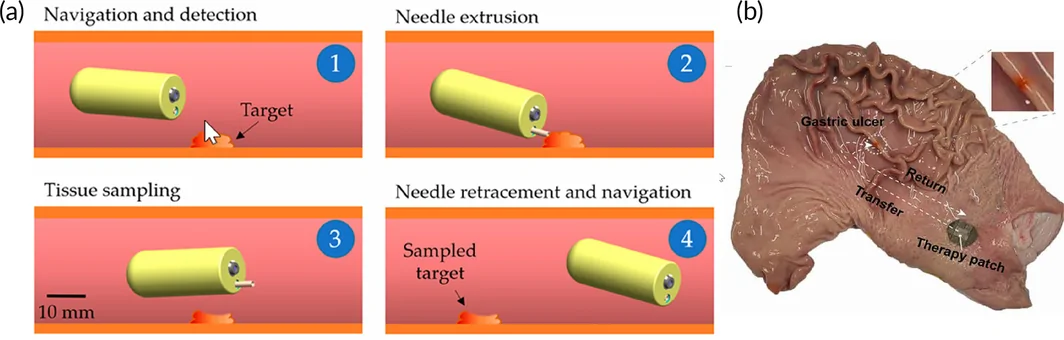
Minimally invasive robotic systems for gastrointestinal drug delivery, biopsy, and mucosal repair. (a) Proposed applications for the intestinal puncture biopsy capsule. Reproduced under terms of the CC‐BY license.^102^ Copyright 2020, Manh Cuong Hoang, published by MDPI. (b) The entire process of a soft robotic patch for treating gastric ulcers. Reproduced under terms of the CC‐BY license.^104^ Copyright 2022, Yue Dong, published by The American Association for the Advancement of Science.

#### 5.2.3 Mucosal repair and in situ tissue regeneration

If damage to the gastrointestinal mucosa is not repaired promptly, prolonged exposure to a highly acidic environment can easily lead to further deterioration. To address this challenge, Dong et al. developed a magnetic soft robot integrated with a therapeutic film (Figure 10b). By utilizing multimodal locomotion—including tumbling and flipping—to navigate the folds of the stomach, the robot successfully positioned the protective patch precisely over the ulcer surface, achieving rapid physical sealing and isolation of the lesion.^104^

### 5.3 Cancer treatment

Despite significant advances in clinical cancer treatment, the invasiveness of surgical resection and the systemic side effects of radiation and chemotherapy remain insurmountable obstacles. In particular, the dense extracellular matrix (ECM) and high interstitial fluid pressure (IFP) within solid tumors create natural physical barriers to passive drug diffusion, making it difficult for therapeutic agents to penetrate deep into the tissue. To address this challenge, magnetically controlled microrobots, leveraging their ability to navigate and penetrate complex physiological environments, are reshaping the paradigm of precision cancer therapy.

#### 5.3.1 Magnetothermal and photothermal physical therapy

In the face of clinical challenges such as resistance to traditional chemotherapy drugs and systemic toxicity, physical hyperthermia using microrobots converts magnetic or light energy into thermal energy to directly induce thermal ablation of tumor cells, offering a new approach to precision cancer therapy that requires little to no medication.

To address the challenge of balancing deep penetration with efficient heat generation in magnetothermal therapy, Xing et al. developed the TriMag microrobot using two‐photon 3D printing technology.^105^ This system combines SPIONs for imaging with hard magnetic particles for heat generation; it not only enables precise magnetic control but also allows for radiation‐free real‐time tracking within deep tissues via magnetic particle imaging (MPI) technology. In the field of photothermal therapy, the application of novel two‐dimensional materials has significantly improved energy conversion efficiency. Yang et al. have developed biodegradable MXene‐based magnetic microrobots.^106^ Benefiting from the excellent photothermal properties of Ti_3_C_2_ MXene, this robot can rapidly heat up under near‐infrared irradiation, enabling targeted thermal ablation of tumor cells (Figure 11a).

**FIGURE 11:**
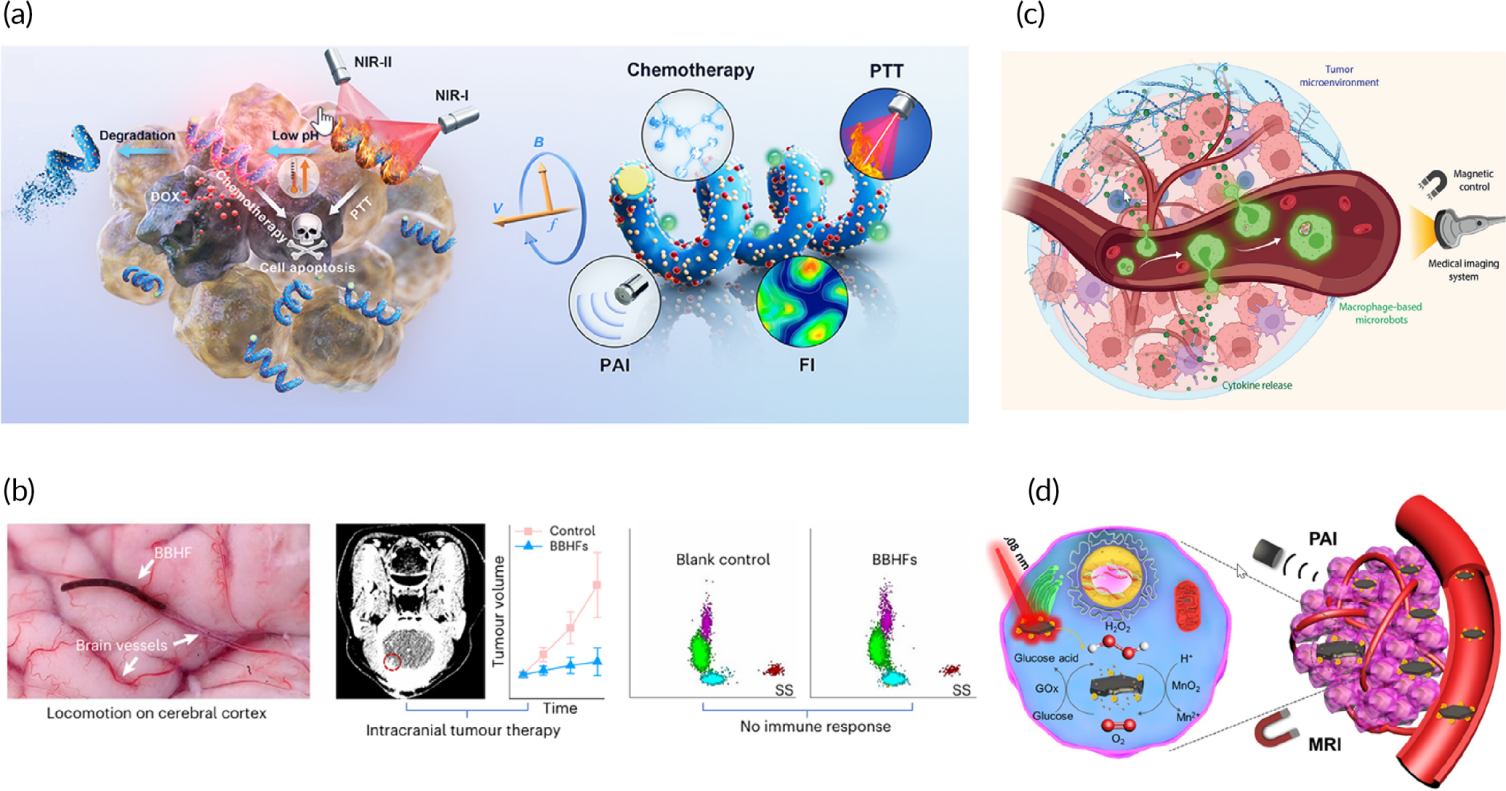
Magnetic robotic and biohybrid platforms for multimodal cancer therapy, including chemo‐photothermal therapy, intracranial tumor treatment, cell‐based immunotherapy, and starvation therapy. (a) Schematic illustration of MXBOTs@DOX for motion control, synergistic chemo‐thermo‐phototherapy, imaging, and biodegradation. Reproduced with permission.^106^ Copyright 2024, American Chemical Society. (b) The chart shows BBHF's ability to treat intracranial tumors without a significant immune response. Reproduced with permission.^109^ Copyright 2025, Springer Nature. (c) Magnetically driven macrophage‐based microrobots for targeted cancer therapy. Reproduced under terms of the CC‐BY license.^110^ Copyright 2024, Nihal Olcay Dogan, published by John Wiley and Sons. (d) Schematic diagram of MNS‐GOx for magnetic resonance (MR)/photoacoustic (PA) dual‐modal imaging guided self‐oxygenation/hyperthermia dually enhanced starvation cancer therapy. Reproduced under terms of the CC‐BY license.^114^ Copyright 2020, Ting He, published by Ivyspring International Publisher.

#### 5.3.2 Localized chemotherapy and immunotherapy

To address the clinical challenges posed by the high systemic toxicity and low intratumoral penetration of traditional chemotherapy drugs, as well as the low response rates of immunotherapy in solid tumors, the use of microrobots to deliver high‐concentration localized chemotherapy and precise immune activation is a key strategy for reducing systemic side effects and overcoming tumor resistance.

In the field of localized chemotherapy, researchers have focused on addressing the challenge of drug penetration into the core of tumors. To tackle the dense hypoxic microenvironment within solid tumors, Felfoul et al. innovatively utilized magnetotactic bacteria as carriers. By leveraging the bacteria's natural chemotaxis, they guided drug‐loaded liposomes to actively penetrate deep into the hypoxic necrotic regions of colorectal tumors, achieving a targeted accumulation effect superior to that of passive nanomedicines.^107^ To address the issue of chemotherapy resistance in malignant osteosarcoma, Tao et al. designed a magnetically driven hydrogel microrobot capable of delivering cell cycle inhibitors (such as Ro‐3306) to enhance tumor sensitivity to chemotherapy through a synthetic lethal strategy.^108^ For intracranial tumors with more complex anatomical structures, Wang et al. developed a biohybrid hydrogel fiber robot prepared from the patient's own blood (Figure 11b). This robot not only possesses excellent immune evasion capabilities but can also navigate through cerebrospinal fluid to deep lesions under real‐time X‐ray fluoroscopy guidance for targeted drug delivery.^109^

In immunotherapy, the key challenge lies in enhancing the infiltration and survival of immune effector cells within tumor tissue. By attaching magnetic Janus particles to macrophages (Figure 11c), Dogan et al. not only achieved long‐distance active transport under magnetic field control but also utilized photoacoustic imaging to monitor the robots' positions in real time, ensuring that the macrophages maintained their antitumor (M1‐type) activity and exerted their immune‐killing effects upon reaching the tumor site.^110^

#### 5.3.3 Mechanical destruction and starvation therapy

In addition to traditional drug delivery, magnetic microrobots can also use physical forces generated by magnetic fields to directly disrupt tumor structures or cut off the tumor's energy supply through metabolic interference, offering a novel, non‐pharmacological strategy for treating drug‐resistant solid tumors.

In the field of mechanical destruction, researchers are focusing on using the dynamic response of magnetic carriers to physically ablate cancer cells. Kim et al. pioneered the development of biofunctionalized microdisks with a spin‐vortex magnetic state; these microdisks generate mechanical oscillatory forces under a low‐frequency alternating magnetic field, capable of directly physically disrupting cancer cell membranes and specifically inducing apoptosis.^111^ To address the disruption of intracellular structures, Shen et al. designed a cluster of smart nanomotors capable of generating adaptive vortices in response to a rotating magnetic field. This cluster can enter cellular lysosomes and use the fluid shear forces generated by high‐speed rotation to disrupt the lysosomal membrane, thereby activating apoptotic signaling pathways.^112^ In addition, regarding the matrix barrier in the tumor microenvironment, Lopez et al. demonstrated that ultrafine iron oxide nanoparticles can generate in situ mechanical torque under a low‐frequency rotating magnetic field, effectively killing cancer‐associated fibroblasts by disrupting lysosomes, thereby dismantling the matrix environment that supports tumor growth.^113^

In the field of starvation therapy, the use of functionalized micro‐ and nanorobots to deplete glucose in the tumor microenvironment has emerged as an effective metabolic therapeutic approach. To address the oxygen dependency of traditional enzyme‐catalyzed reactions, He et al. developed a self‐oxygenating system based on manganese dioxide (MnO_2_) nanosheets. This system utilizes glucose oxidase (GOx) to consume glucose and produce H_2_O_2_, which is then converted into oxygen by MnO_2_, thereby enhancing starvation therapy while improving the efficiency of photothermal therapy (Figure 11d).^114^

Furthermore, to exploit the hypoxic microenvironment induced by starvation, Shao et al. developed a biomimetic nanoreactor designed to mimic the surface of cancer cells. This nanoreactor starves tumor cells to death via GOx while simultaneously utilizing the resulting severe hypoxic environment to activate the co‐loaded hypoxic prodrug (AQ4N), thereby achieving a cascading, amplified cytotoxic effect through starvation‐chemotherapy.^115^

### 5.4 Other scenarios

In addition to their widespread use in vascular, gastrointestinal, and cancer treatments, magnetically controlled microrobots are opening up new frontiers in clinical applications within ophthalmic surgery, tissue engineering, and anti‐infective therapy, owing to their non‐invasive intervention capabilities within enclosed cavities and their ability to operate with subcellular precision. These applications typically demand extremely high precision and are subject to severely limited operating space. The remote‐controlled nature of microrobots effectively fills the gaps left by traditional microsurgery.

#### 5.4.1 Advanced ophthalmic surgery

The interior of the eyeball is filled with a viscous vitreous humor, whose dense collagen network forms a natural barrier to drug diffusion. Traditional intraocular injections struggle to achieve targeted delivery to specific regions of the retina, but microrobots hold promise for solving this challenge. Inspired by the lubricious surface of the pitcher plant, Wu et al. developed a slippery micro‐propeller with a perfluorocarbon coating (Figure 12a). This special liquid coating significantly reduces the adhesion to the surrounding biomolecules. Driven by a rotating magnetic field, this swarm of micro‐propellers can easily penetrate the dense vitreous matrix of a pig's eyeball and reach the optic disc region near the retina in a controlled manner.^116^

**FIGURE 12:**
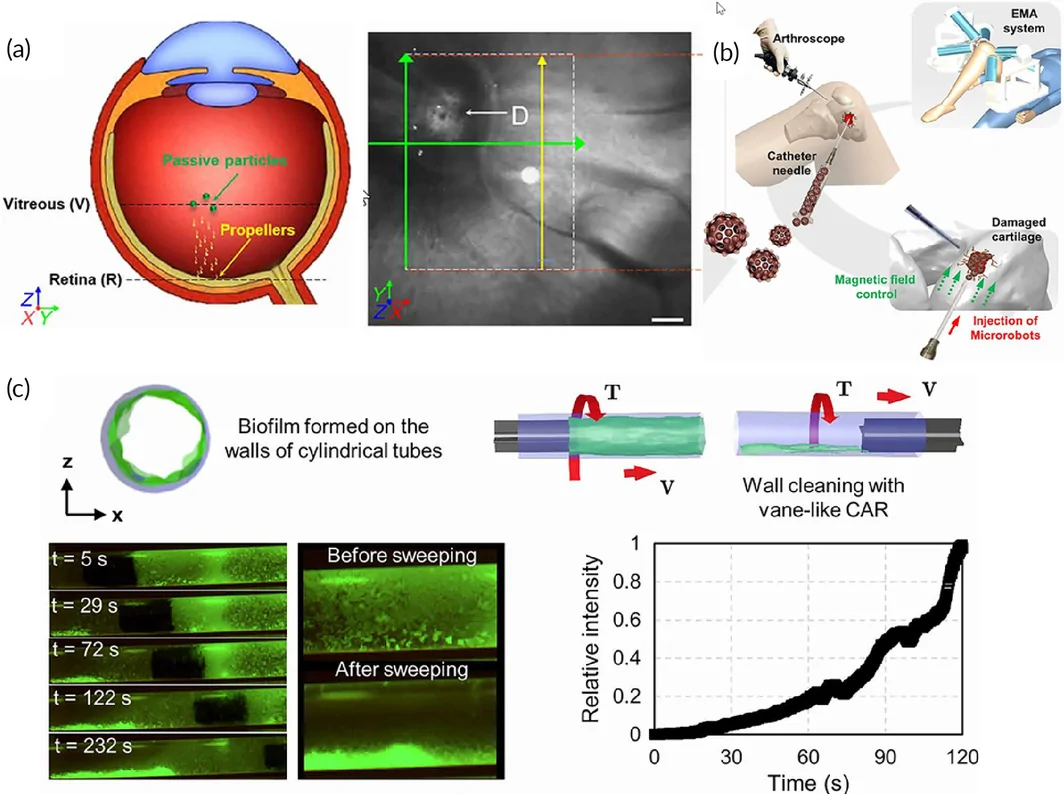
Applications in ophthalmic delivery, cartilage regeneration, and biofilm eradication. (a) The process by which a slippery micropropeller moves from the vitreous toward the retina. Reproduced under terms of the CC‐BY license.^116^ Copyright 2018, Zhiguang Wu, published by The American Association for the Advancement of Science. (b) The process of knee cartilage regeneration using a magnetic microrobot‐mediated MSC delivery system. Reproduced with permission.^118^ Copyright 2020, The American Association for the Advancement of Science. (c) Vane CARs remove biofilms from glass tube walls and eliminate debris using a magnetic field. Reproduced with permission.^121^ Copyright 2019, The American Association for the Advancement of Science.

#### 5.4.2 Tissue engineering and regenerative medicine

Repairing damaged cartilage or neural tissue requires the precise delivery and engraftment of therapeutic cells or scaffolds at the site of the lesion. To address the challenges of low cell retention rates and poor targeting in traditional stem cell transplantation, Jeon et al. developed a magnetic microrobot platform with a 3D porous structure. Stem cells loaded onto this platform can effectively proliferate and differentiate into astrocytes, oligodendrocytes, and neurons upon reaching the target area.^117^ In the application of articular cartilage repair, Go et al. further proposed a microrobotic system based on human adipose‐derived mesenchymal stem cells (Figure 12b). This system enables the long‐term in situ colonization of cell carriers via magnetic anchors, significantly promoting cartilage regeneration in vivo models.^118^

In the field of neural tissue engineering, microrobots are used to restore the function of disrupted neural networks. Kim et al. designed a magnetic microrobot loaded with neurons that can be precisely positioned between two separated neural populations to act as a microbridge. By establishing a physical connection, it induces the growth of neural processes and successfully restores the transmission of action potential signals between the neural clusters.^119^ For the structural reconstruction of long‐segment peripheral nerve defects, Hong et al. utilized magnetic fields to align iron oxide nanoparticles to construct an anisotropic fibrin nanofiber hydrogel. This uniform nanofiber magnetic scaffold significantly enhances the expression of nerve growth factor (NGF) and vascular endothelial growth factor (VEGF), thereby accelerating peripheral nerve regeneration and functional recovery.^120^

#### 5.4.3 Biofilm removal and infection prevention

Bacterial biofilms on the surfaces of medical implants (such as dental implants and stents) exhibit extremely high levels of antibiotic resistance and are difficult to completely eradicate with antibiotics. Hwang et al. have developed a dual‐function catalytic antimicrobial robot (CARs).^121^ This robot is constructed from iron oxide nanoparticles. On the one hand, it utilizes the peroxidase‐like activity of iron oxide to catalyze hydrogen peroxide, generating high concentrations of hydroxyl radicals that oxidize and kill bacteria exposed by mechanical disruption; on the other hand, it uses a magnetic field to form plow‐like aggregates that mechanically scrape away the extracellular polymeric matrix of the biofilm. Experiments demonstrate that this synergistic strategy of mechanical removal and chemical disinfection can efficiently eliminate stubborn biofilms from glass tube walls and complex surfaces, and can remove detached biofilm fragments via a magnetic field to prevent secondary infection (Figure 12c).

## 6 CONCLUSION

This review provides a comprehensive synthesis of the research landscape regarding magnetic microrobots in medical applications. As a transformative paradigm in minimally invasive medicine, magnetic microrobots circumvent the inherent physical limitations of traditional tethered instruments, such as fixed dimensions, mechanical stiffness, and limited accessibility, offering a novel solution for high‐precision diagnostics and therapeutics within deep‐seated and delicate anatomical regions.

In terms of locomotion control, magnetic microrobots have evolved from early single‐mode translational systems into multimodal systems with exceptional environmental adaptability. Current research underscores the necessity of tailoring propulsion strategies to specific physiological terrains: translational motion utilizing magnetic gradient forces is suitable for long‐distance transport across wide gaps; spiral swimming mimicking microorganisms is an effective means of overcoming low‐Reynolds‐number viscous resistance in blood vessels; while tumbling motion based on frictional coupling and jumping motion driven by the release of elastic energy have demonstrated exceptional performance in adhering to gastrointestinal surfaces and traversing macroscopic fold obstacles, respectively.

Regarding material selection, the field is witnessing a transition from structural simplicity toward functionalized, intelligent integration, with the central challenge being the trade‐off between magnetic performance and biocompatibility. While hard magnetic materials endow robots with excellent three‐dimensional mobility, they present challenges related to the encapsulation of heavy metals; soft magnetic materials, due to their on‐demand magnetization and integrated diagnostic and therapeutic capabilities, have emerged as the preferred choice. At the same time, matrix materials have evolved from inert elastomers to bioactive materials capable of environmental responsiveness and biodegradability.

Advanced manufacturing technologies represent the critical bridge for translating microrobot designs into functional devices. To address the multi‐scale characteristics of micro‐robots—ranging from the micro‐ and nano‐scales to the millimeter scale—and the need for heterogeneous material integration, a diversified manufacturing system has emerged, centered on additive manufacturing, microfluidics, and self‐assembly. Among these, additive manufacturing (TPP/DIW) has overcome the geometric limitations of traditional microfabrication, establishing itself as the mainstream approach for constructing complex three‐dimensional structures and in‐situ magnetic programming; microfluidic technology, through its precise manipulation of multiphase flows, has resolved the challenges of high‐throughput, low‐cost manufacturing for hydrogel‐based drug‐ or cell‐carrying robots, serving as a key driver for scaling biomedical applications; meanwhile, template‐assisted deposition and self‐assembly technologies have effectively filled the gap in precision manufacturing at the nanoscale and the construction of reconfigurable clusters.

From the perspective of clinical translation, magnetic microrobots are poised to redefine existing medical interventions. In vascular surgery, their ability to navigate against blood flow and perform intelligent embolization is expected to reduce systemic risks in stroke and cancer treatment. In gastrointestinal diagnostics and treatment, active capsule endoscopy and multifunctional magnetic soft robots for targeted delivery, biopsy, and mucosal repair are facilitating a transition from passive observation to active therapy. In cancer treatment, integrated strategies combining magnetic/photothermal ablation, immune activation, and mechanical starvation therapy have opened new pathways for conquering drug‐resistant solid tumors with minimal or no drug use.

Despite substantial advancements in magnetic microrobots, several major challenges persist that impede their seamless integration into clinical practice. First and foremost are concerns regarding biocompatibility and metabolic fate. Currently, most high‐performance robots predominantly utilize metallic components such as nickel and neodymium; the potential for long‐term toxicity remains a significant regulatory bottleneck, notwithstanding progress in protective surface functionalization. Secondly, achieving real‐time, high‐resolution imaging of deep‐seated tissues remains elusive. Existing optical modalities suffer from limited penetration depths in biological tissues, while clinical standards such as MRI and CT fail to balance the requirements for real‐time tracking of micrometer‐scale robots in terms of imaging speed and spatial resolution. Lastly, the implementation of swarm intelligence within complex dynamic in vivo environments poses significant control difficulties. The internal physiological environment is characterized by pulsating fluids, soft tissue deformation, and non‐Newtonian rheological properties. While the functional capacity of an individual agent is inherently limited, coordinating large‐scale swarms remains fraught with challenges related to formation stability and disintegration.

In summary, magnetic microrobot technology has reached a pivotal inflection point, transitioning from laboratory research and development toward clinical translation. Through synergistic, cross‐disciplinary collaboration—bridging materials science, medical imaging, and clinical medicine—these micro‐ and nano‐scale intelligent systems are poised to transcend the physical constraints of conventional minimally invasive instruments. Ultimately, they represent the vanguard of a new generation of theranostic platforms, destined to reshape the future of precision medicine by facilitating unprecedented access to the most distal and delicate regions of the human body.

## AUTHOR CONTRIBUTIONS

**Gongxin Li:** Conceptualization; methodology; project administration; writing – review and editing; supervision; investigation; funding acquisition. **Yuting Mao:** Conceptualization; methodology; formal analysis; data curation; writing – original draft. **Fei Liu:** Methodology; validation; investigation; data curation. **Fangmei An:** Methodology; formal analysis; investigation; validation. **Qiang Zhan:** Conceptualization; methodology; supervision; resources; writing – review and editing.

## CONFLICT OF INTEREST STATEMENT

The authors declare no conflicts of interest.

## Data Availability

Data sharing not applicable to this article as no datasets were generated or analysed during the current study.

## References

[1] Zhang D , Gorochowski TE , Marucci L , et al. Advanced medical micro‐robotics for early diagnosis and therapeutic interventions. Front Rob AI. 2023;9:1086043. 10.3389/frobt.2022.1086043 PMC9871318 36704240

[2] Bozuyuk U , Wrede P , Yildiz E , Sitti M . Roadmap for clinical translation of mobile microrobotics. Adv Mater. 2024;36(23):2311462. 10.1002/adma.202311462 38380776

[3] Li J , Esteban‐Fernández de Ávila B , Gao W , et al. Micro/nanorobots for biomedicine: delivery, surgery, sensing, and detoxification. Sci Robot. 2017;2(4):eaam6431. 10.1126/scirobotics.aam6431 PMC6759331 31552379

[4] Zhang Y , Li J , Yin L , et al. Magnetically guided microrobots for targeted drug delivery. Adv Healthc Mater. 2026;15(14):e05942. 10.1002/adhm.202505942 41568887

[5] Nelson BJ , Kaliakatsos IK , Abbott JJ . Microrobots for minimally invasive medicine. Annu Rev Biomed Eng. 2010;12(1):55‐85. 10.1146/annurev-bioeng-010510-103409 20415589

[6] Sitti M , Ceylan H , Hu W , et al. Biomedical applications of untethered mobile milli/microrobots. Proc IEEE. 2015;103(2):205‐224. 10.1109/JPROC.2014.2385105 PMC5063027 27746484

[7] Zhou J , Li M , Li N , Zhou Y , Wang J , Jiao N . System integration of magnetic medical microrobots: from design to control. Front Rob AI. 2023;10:1330960. 10.3389/frobt.2023.1330960 PMC10758462 38169802

[8] Zhou H , Mayorga‐Martinez CC , Pané S , Zhang L , Pumera M . Magnetically driven micro and Nanorobots. Chem Rev. 2021;121(8):4999‐5041. 10.1021/acs.chemrev.0c01234 PMC8154323 33787235

[9] Sun J , Jia X , Xue J , et al. Tumbler‐inspired oral microrobot system with dual modules for on‐demand gastric drug delivery. ACS Appl Mater Interfaces. 2026;18(10):14674‐14684. 10.1021/acsami.5c24943 41782274

[10] Xin C , Zhang C , Dai Y , et al. Femtosecond laser writing of stepwise detachable and biodegradable microrobots for adjustable locomotion and drug delivery. Adv Mater. 2026;38:e75013. 10.1002/adma.75013 42750394

[11] Zhong S , Hou Y , Zheng Z , et al. Adaptive shared cascade navigation control of magnetic microrobots in unstructured dynamic environments. IEEE Trans Cyber. 2026;56:3753‐3766. 10.1109/TCYB.2026.3658831 41666053

[12] Hu W , Lum GZ , Mastrangeli M , Sitti M . Small‐scale soft‐bodied robot with multimodal locomotion. Nature. 2018;554(7690):81‐85. 10.1038/nature25443 29364873

[13] Martel S , Mathieu J‐B , Felfoul O , et al. Automatic navigation of an untethered device in the artery of a living animal using a conventional clinical magnetic resonance imaging system. Appl Phys Lett. 2007;90(11):114105.

[14] Kummer MP , Abbott JJ , Kratochvil BE , Borer R , Sengul A , Nelson BJ . OctoMag: an electromagnetic system for 5‐DOF wireless micromanipulation. IEEE Trans Robot. 2010;26(6):1006‐1017.

[15] Pawashe C , Floyd S , Sitti M . Modeling and experimental characterization of an untethered magnetic micro‐robot. Int J Robot Res. 2009;28(8):1077‐1094.

[16] Ullrich F , Bergeles C , Pokki J , et al. Mobility experiments with microrobots for minimally invasive intraocular surgery. Invest Ophthalmol Vis Sci. 2013;54(4):2853‐2863. 10.1167/iovs.13-11825 23518764

[17] Chen Z , Wang Y , Chen H , et al. A magnetic multi‐layer soft robot for on‐demand targeted adhesion. Nat Commun. 2024;15(1):644. 10.1038/s41467-024-44995-9 PMC10799857 38245517

[18] Chen B , Wu H , Chen Y , et al. Cooperative magnetic interfacial microrobot couple for versatile non‐contact biomedical applications. Adv Mater. 2025;37(12):2417416. 10.1002/adma.202417416 39967348

[19] Cao H , Xing L , Hu J , Mo H , Sun D . Automated control of microparticle swarm in a rotating gradient‐based magnetic field. IEEE Trans Autom Sci Eng. 2025;22:2946‐2955.

[20] Landers FC , Hertle L , Pustovalov V , et al. Clinically ready magnetic microrobots for targeted therapies. Science. 2025;390(6774):710‐715. 10.1126/science.adx1708 41231973

[21] Abbott JJ , Peyer KE , Lagomarsino MC , et al. How should microrobots swim? Int J Robot Res. 2009;28(11–12):1434‐1447.

[22] Xu T , Yu J , Yan X , Choi H , Zhang L . Magnetic actuation based motion control for microrobots: an overview. Micromachines. 2015;6(9):1346‐1364.

[23] Dreyfus R , Baudry J , Roper ML , Fermigier M , Stone HA , Bibette J . Microscopic artificial swimmers. Nature. 2005;437(7060):862‐865. 10.1038/nature04090 16208366

[24] Huang C , Xu T , Liu J , Manamanchaiyaporn L , Wu X . Visual servoing of miniature magnetic film swimming robots for 3‐D arbitrary path following. IEEE Robot Autom Lett. 2019;4(4):4185‐4191.

[25] Zhang L , Abbott JJ , Dong L , Kratochvil BE , Bell D , Nelson BJ . Artificial bacterial flagella: fabrication and magnetic control. Appl Phys Lett. 2009;94(6):064107.

[26] Wu D , Wang X , Li R , et al. Femtosecond laser‐assisted printing of hard magnetic microrobots for swimming upstream in subcentimeter‐per‐second blood flow. Sci Adv. 2025;11(27):eadw1272. 10.1126/sciadv.adw1272 PMC12227040 40614197

[27] Ceylan H , Yasa IC , Yasa O , Tabak AF , Giltinan J , Sitti M . 3D‐printed biodegradable microswimmer for theranostic cargo delivery and release. ACS Nano. 2019;13(3):3353‐3362. 10.1021/acsnano.8b09233 PMC6728090 30742410

[28] Xu H , Medina‐Sánchez M , Maitz MF , Werner C , Schmidt OG . Sperm micromotors for cargo delivery through flowing blood. ACS Nano. 2020;14(3):2982‐2993. 10.1021/acsnano.9b07851 32096976

[29] Alapan Y , Bozuyuk U , Erkoc P , Karacakol AC , Sitti M . Multifunctional surface microrollers for targeted cargo delivery in physiological blood flow. Sci Robot. 2020;5(42):eaba5726. 10.1126/scirobotics.aba5726 33022624

[30] Bi C , Guix M , Johnson B , Jing W , Cappelleri D . Design of microscale magnetic tumbling robots for locomotion in multiple environments and complex terrains. Micromachines. 2018;9(2):68. 10.3390/mi9020068 PMC6187462 30393344

[31] Niedert EE , Bi C , Adam G , et al. A tumbling magnetic microrobot system for biomedical applications. Micromachines. 2020;11(9):861. 10.3390/mi11090861 PMC7569920 32957563

[32] Sehyuk Y , Sitti M . Shape‐programmable soft capsule robots for semi‐implantable drug delivery. IEEE Trans Robot. 2012;28(5):1198‐1202.

[33] Wu Y , Dong X , Kim J‐k , Wang C , Sitti M . Wireless soft millirobots for climbing three‐dimensional surfaces in confined spaces. Sci Adv. 2022;8(21):eabn3431. 10.1126/sciadv.abn3431 PMC9140972 35622917

[34] Hou Y , Zhong S , Zheng Z , et al. Magnetic shaftless propeller millirobot with multimodal motion for small‐scale fluidic manipulation. Cyborg Bionic Syst. 2025;6:235. 10.34133/cbsystems.0235 PMC11903028 40078330

[35] Sun Y , Zhang W , Gu J , et al. Magnetically driven capsules with multimodal response and multifunctionality for biomedical applications. Nat Commun. 2024;15(1):1839. 10.1038/s41467-024-46046-9 PMC10904804 38424039

[36] Campbell JF , Kaya HK . How and why a parasitic nematode jumps. Nature. 1999;397(6719):485‐486.

[37] Soto F , Karshalev E , Zhang F , et al. Smart materials for microrobots. Chem Rev. 2021;122(5):5365‐5403. 10.1021/acs.chemrev.0c00999 33522238

[38] Wang Y , Du X , Zhang H , et al. Amphibious miniature soft jumping robot with on‐demand in‐flight maneuver. Adv Sci. 2023;10(18):2207493. 10.1002/advs.202207493 PMC10288233 37097734

[39] Tang D , Zhang C , Pan C , et al. Bistable soft jumper capable of fast response and high takeoff velocity. Sci Robot. 2024;9(93):eadm8484. 10.1126/scirobotics.adm8484 39167670

[40] Kim Y , Zhao X . Magnetic soft materials and robots. Chem Rev. 2022;122(5):5317‐5364. 10.1021/acs.chemrev.1c00481 PMC9211764 35104403

[41] Xu R , Xu Q . A survey of recent developments in magnetic microrobots for micro‐/nano‐manipulation. Micromachines. 2024;15(4):468. 10.3390/mi15040468 PMC11052276 38675279

[42] Ebrahimi N , Bi C , Cappelleri DJ , et al. Magnetic actuation methods in bio/soft robotics. Adv Funct Mater. 2020;31(11):2005137.

[43] Kittel C . Physical theory of ferromagnetic domains. Rev Mod Phys. 1949;21(4):541‐583.

[44] Rikken RSM , Nolte RJM , Maan JC , van Hest JCM , Wilson DA , Christianen PCM . Manipulation of micro‐ and nanostructure motion with magnetic fields. Soft Matter. 2014;10(9):1295‐1308. 10.1039/c3sm52294f 24652392

[45] Chen J , Jin D , Wang Q , Ma X . Programming ferromagnetic soft materials for miniature soft robots: design, fabrication, and applications. J Mater Sci Technol. 2025;219:271‐287.

[46] Sarriegui G , Martin JM , Ipatov M , Zhukov AP , Gonzalez J . Magnetic properties of NdFeB alloys obtained by gas atomization technique. IEEE Trans Magn. 2018;54(11):1‐5.

[47] Ma B , Herchenroeder J , Smith B , et al. Recent development in bonded NdFeB magnets. J Magn Magn Mater. 2002;239(1–3):418‐423.

[48] Taylor CR , Nott JK , Ratnasena NH , Cohen JM , Herr HM . Non‐pyrogenicity and biocompatibility of parylene‐coated magnetic bead implants. Front Bioeng Biotechnol. 2024;12:1290453. 10.3389/fbioe.2024.1290453 PMC10912624 38444650

[49] Iacovacci V , Naselli I , Salgarella AR , Clemente F , Ricotti L , Cipriani C . Stability and in vivo safety of gold, titanium nitride and parylene C coatings on NdFeB magnets implanted in muscles towards a new generation of myokinetic prosthetic limbs. RSC Adv. 2021;11(12):6766‐6775. 10.1039/d0ra07989h PMC8694929 35423178

[50] Li J , Fan L , Li Y , et al. Development of cell‐carrying magnetic microrobots with bioactive nanostructured titanate surface for enhanced cell adhesion. Micromachines. 2021;12(12):1572. 10.3390/mi12121572 PMC8707319 34945424

[51] Kim D‐i , Lee H , Kwon S‐h , Choi H , Park S . Magnetic nano‐particles retrievable biodegradable hydrogel microrobot. Sens Actuators B Chem. 2019;289:65‐77.

[52] Song W , Li L , Liu X , et al. Hydrogel microrobots for biomedical applications. Front Chem. 2024;12:1416314. 10.3389/fchem.2024.1416314 PMC11150770 38841335

[53] Kim J , Park H , Yoon C . Advances in biodegradable soft robots. Polymers (Basel). 2022;14(21):4574. 10.3390/polym14214574 PMC9658808 36365570

[54] Ren A , Hu J , Qin C , et al. Oral administration microrobots for drug delivery. Bioact Mater. 2024;39:163‐190. 10.1016/j.bioactmat.2024.05.005 PMC11130999 38808156

[55] Aybar Tural G , Ozgen G , Bozuyuk U , et al. Droplet‐based microfluidic production of soft alginate microrobots for magnetically targeted cargo delivery. Int J Pharm. 2025;685:126170. 10.1016/j.ijpharm.2025.126170 40983120

[56] Wang X , Li W , Lou Q , Li W , Zhang L , Weitz DA . Single‐cell microgel microrobot for targeted and imaging‐guided cell therapy. CCS Chem. 2025;7(12):3732‐3744.

[57] Veloso SRS , Vázquez‐González M , Ribeiro MO , et al. Chitosan/alginate polyelectrolyte magnetic gel nanoarchitectonics with tunable mechanical properties for magnetic hyperthermia and sustained release of 5‐fluorouracil. ACS Appl Mater Interfaces. 2025;17(45):61731‐61741. 10.1021/acsami.5c15863 PMC12616591 41148171

[58] Wang H , Song X , Xiong J , Cheang UK . Fabrication of bilayer magnetically actuated L‐shaped microrobot based on chitosan via photolithography. Polymers. 2022;14(24):5509. 10.3390/polym14245509 PMC9784805 36559876

[59] Wei J , Liu Z , Li M , et al. A biodegradable magnetic microrobot based on alginate/GelMA microcapsules for delivery of chondroitin sulfate for cartilage tissue repair. Int J Biol Macromol. 2025;321:146296. 10.1016/j.ijbiomac.2025.146296 40738438

[60] Maia MV , Egito EST , Sapin‐Minet A , et al. Fibroin‐hybrid systems: current advances in biomedical applications. Molecules. 2025;30(2):328. 10.3390/molecules30020328 PMC11767523 39860198

[61] Protsak IS , Morozov YM . Fundamentals and advances in stimuli‐responsive hydrogels and their applications: a review. Gels. 2025;11(1):30. 10.3390/gels11010030 PMC11765116 39852001

[62] Siebenmorgen C , Wang C , Navarro LB , et al. Minimally designed thermo‐magnetic dual responsive soft robots for complex applications. J Mater Chem B. 2024;12(22):5339‐5349. 10.1039/d3tb02839a PMC11152039 38597898

[63] Marechal L , Balland P , Lindenroth L , Petrou F , Kontovounisios C , Bello F . Toward a common framework and database of materials for soft robotics. Soft Rob. 2021;8(3):284‐297. 10.1089/soro.2019.0115 32589507

[64] Porte E , Eristoff S , Agrawala A , Kramer‐Bottiglio R . Characterization of temperature and humidity dependence in soft elastomer behavior. Soft Rob. 2024;11(1):118‐130. 10.1089/soro.2023.0004 PMC10880277 37669451

[65] Kalashnikov N , Thareja R , Vorstenbosch J . Tissue‐material characteristics determine fibrous encapsulation dynamics across common polymeric surgical biomaterials in a tissue‐specific mouse foreign body response model. J Biomed Mater Res A. 2026;114(6):e70096. 10.1002/jbm.a.70096 42206683

[66] Krpovic S , Dam‐Johansen K , Skov AL . Importance of Mullins effect in commercial silicone elastomer formulations for soft robotics. J Appl Polym Sci. 2021;138(19):50380.

[67] Makadia H , Siegel S . Poly lactic‐co‐glycolic acid (PLGA) as biodegradable controlled drug delivery carrier. Polymers (Basel). 2011;3(3):1377‐1397. 10.3390/polym3031377 PMC3347861 22577513

[68] Li J , Liang Z , Li T , Wan Q , Huang X , Kan Q . A comprehensive review on 4D printing and applications of thermo‐induced shape memory polymers. Int J Smart Nano Mater. 2025;16(1):24‐83.

[69] Kortman VG , de Vries E , Jovanova J , Sakes A . Magnetic stimulation for programmed shape morphing: review of four‐dimensional printing, challenges and opportunities. 3D Print Addit Manuf. 2024;11(3):977‐993. 10.1089/3dp.2023.0198 PMC11442361 39359596

[70] Sun M , Tian C , Mao L , et al. Reconfigurable magnetic slime robot: deformation, adaptability, and multifunction. Adv Funct Mater. 2022;32(26):2112508.

[71] Wang X , Guo R , Liu J . Liquid metal based soft robotics: materials, designs, and applications. Adv Mater Technol. 2019;4(2):1800549.

[72] Wu Y , Ye D , Shan Y , et al. Edible and nutritive electronics: materials, fabrications, components, and applications. Adv Mater Technol. 2020;5(10):2000100.

[73] Baumgartner M , Hartmann F , Drack M , et al. Resilient yet entirely degradable gelatin‐based biogels for soft robots and electronics. Nat Mater. 2020;19(10):1102‐1109. 10.1038/s41563-020-0699-3 32541932

[74] Li R , Jiang M , Liu B , et al. High‐performance magnetic metal microrobot prepared by a two‐photon polymerization and sintering method. Lab Chip. 2024;24(4):832‐842. 10.1039/d3lc01084h 38235769

[75] Wang J , Zheng J , Zhang C , et al. 4D‐printed microrobots with synergistic integration of magnetic actuation, diffractive optical sensing, and pH‐adaptive morphing. Adv Funct Mater. 2025;36:e18838.

[76] Kim Y , Yuk H , Zhao R , Chester SA , Zhao X . Printing ferromagnetic domains for untethered fast‐transforming soft materials. Nature. 2018;558(7709):274‐279. 10.1038/s41586-018-0185-0 29899476

[77] He T , Yang Y , Chen X‐B . Preparation, stimulus–response mechanisms and applications of micro/nanorobots. Micromachines (Basel). 2023;14(12):2253. 10.3390/mi14122253 PMC10745970 38138422

[78] Fournier‐Bidoz S , Arsenault AC , Manners I , et al. Synthetic self‐propelled nanorotors. Chem Commun. 2005;4:441‐443. 10.1039/b414896g 15654363

[79] Alcântara CCJ , Kim S , Lee S , et al. 3D fabrication of fully iron magnetic microrobots. Small. 2019;15(16):1805006. 10.1002/smll.201805006 30829003

[80] Chatzipirpiridis G , de Marco C , Pellicer E , et al. Template‐assisted electroforming of fully semi‐hard‐magnetic helical microactuators. Adv Eng Mater. 2018;20(9):1800179.

[81] Whitesides GM . The origins and the future of microfluidics. Nature. 2006;442(7101):368‐373. 10.1038/nature05058 16871203

[82] Shang L , Cheng Y , Zhao Y . Emerging droplet microfluidics. Chem Rev. 2017;117(12):7964‐8040. 10.1021/acs.chemrev.6b00848 28537383

[83] Shah RK , Shum HC , Rowat AC , et al. Designer emulsions using microfluidics. Mater Today. 2008;11(4):18‐27.

[84] Dendukuri D , Pregibon DC , Collins J , Hatton TA , Doyle PS . Continuous‐flow lithography for high‐throughput microparticle synthesis. Nat Mater. 2006;5(5):365‐369. 10.1038/nmat1617 16604080

[85] Yu J , Yang L , Du X , et al. Adaptive pattern and motion control of magnetic microrobotic swarms. IEEE Trans Robot. 2022;38(3):1552‐1570.

[86] Ussia M , Urso M , Oral CM , Peng X , Pumera M . Magnetic microrobot swarms with polymeric hands catching bacteria and microplastics in water. ACS Nano. 2024;18(20):13171‐13183. 10.1021/acsnano.4c02115 PMC11112980 38717036

[87] Zhang J , Ren Z , Hu W , et al. Voxelated three‐dimensional miniature magnetic soft machines via multimaterial heterogeneous assembly. Sci Robot. 2021;6(53):eabf0112. 10.1126/scirobotics.abf0112 PMC7612277 34043568

[88] Chen W , Sun M , Fan X , Xie H . Magnetic/pH‐sensitive double‐layer microrobots for drug delivery and sustained release. Appl Mater Today. 2020;19:100583.

[89] Chen X , Tian C , Zhang H , Xie H . Biodegradable magnetic hydrogel robot with multimodal locomotion for targeted cargo delivery. ACS Appl Mater Interfaces. 2023;15(24):28922‐28932. 10.1021/acsami.3c02703 37283440

[90] Wang J , Zhou Q , Dong Q , et al. Nanoarchitectonic engineering of thermal‐responsive magnetic nanorobot collectives for intracranial aneurysm therapy. Small. 2024;20:2400408. 10.1002/smll.202400408 38709208

[91] Lee S , Kim S , Kim S , et al. A capsule‐type microrobot with pick‐and‐drop motion for targeted drug and cell delivery. Adv Healthc Mater. 2018;7(9):e1700985. 10.1002/adhm.201700985 29460365

[92] Yang L , Zhang T , Huang H , Ren H , Shang W , Shen Y . An On‐Wall‐rotating strategy for effective upstream motion of untethered millirobot: principle, design, and demonstration. IEEE Trans Robot. 2023;39(3):2419‐2428.

[93] Wu Z , Zhang Y , Ai N , Chen H , Ge W , Xu Q . Magnetic Mobile microrobots for upstream and downstream navigation in biofluids with variable flow rate. Adv Intell Syst. 2022;4(7):2100266.

[94] Law J , Du X , Tang W , et al. Navigation of magnetic microrobotic swarms with maintained structural integrity in fluidic flow. IEEE/ASME Trans Mechatron. 2024;29(1):74‐84.

[95] Wang S , Guo X , Xiu W , et al. Accelerating thrombolysis using a precision and clot‐penetrating drug delivery strategy by nanoparticle‐shelled microbubbles. Sci Adv. 2020;6(31):eaaz8204. 10.1126/sciadv.aaz8204 PMC7439573 32832678

[96] Leclerc J , Zhao H , Bao D , et al. In vitro design investigation of a rotating helical magnetic swimmer for combined 3‐D navigation and blood clot removal. IEEE Trans Robot. 2020;36(3):975‐982.

[97] Liu X , Wang L , Xiang Y , et al. Magnetic soft microfiberbots for robotic embolization. Sci Robot. 2024;9(87):eadh2479. 10.1126/scirobotics.adh2479 38381840

[98] Law J , Wang X , Luo M , et al. Microrobotic swarms for selective embolization. Sci Adv. 2022;8(29):eabm5752. 10.1126/sciadv.abm5752 PMC9299543 35857830

[99] Wang B , Chan KF , Yuan K , et al. Endoscopy‐assisted magnetic navigation of biohybrid soft microrobots with rapid endoluminal delivery and imaging. Sci Robot. 2021;6(52):eabd2813. 10.1126/scirobotics.abd2813 34043547

[100] Sehyuk Y , Gultepe E , Gracias DH , et al. Biopsy using a magnetic capsule endoscope carrying, releasing, and retrieving untethered microgrippers. IEEE Trans Biomed Eng. 2014;61(2):513‐521. 10.1109/TBME.2013.2283369 PMC4023810 24108454

[101] Son D , Gilbert H , Sitti M . Magnetically actuated soft capsule endoscope for fine‐needle biopsy. Soft Robot. 2020;7(1):10‐21. 10.1089/soro.2018.0171 31418640

[102] Hoang M , Le V , Nguyen K , et al. A robotic biopsy endoscope with magnetic 5‐DOF locomotion and a retractable biopsy punch. Micromachines (Basel). 2020;11(1):98. 10.3390/mi11010098 PMC7020148 31963402

[103] Xu X , Gao J , Tong D , Zhao Y , Fan X , Ge W . A multifunctional capsule‐like puncture biopsy robot for the gastrointestinal system. Micromachines (Basel). 2025;16(5):589. 10.3390/mi16050589 PMC12113727 40428715

[104] Dong Y , Wang L , Xia N , et al. Untethered small‐scale magnetic soft robot with programmable magnetization and integrated multifunctional modules. Sci Adv. 2022;8(25):eabn8932. 10.1126/sciadv.abn8932 PMC9217092 35731876

[105] Xing L , Cai Y , Zhang Y , et al. TriMag microrobots: 3D‐printed microrobots for magnetic actuation, imaging, and hyperthermia. Adv Mater. 2025;37(45):e19708. 10.1002/adma.202419708 PMC12617050 40787971

[106] Yang M , Xie H , Jiang T , et al. MXBOTs: biodegradable Ti3C2 MXene‐based microrobots for targeted delivery and synergistic chemo‐photothermal therapy. ACS Mater Lett. 2024;6(5):1801‐1810.

[107] Felfoul O , Mohammadi M , Taherkhani S , et al. Magneto‐aerotactic bacteria deliver drug‐containing nanoliposomes to tumour hypoxic regions. Nat Nanotechnol. 2016;11(11):941‐947. 10.1038/nnano.2016.137 PMC6094936 27525475

[108] Tao Y , Li L , Yang X , et al. Magnetic‐driven hydrogel microrobots for promoting osteosarcoma chemo‐therapy with synthetic lethality strategy. Front Chem. 2024;12:1386076. 10.3389/fchem.2024.1386076 PMC11024356 38638876

[109] Wang B , Shen J , Huang C , et al. Magnetically driven biohybrid blood hydrogel fibres for personalized intracranial tumour therapy under fluoroscopic tracking. Nat Biomed Eng. 2025;9(9):1471‐1485. 10.1038/s41551-025-01382-z 40312457

[110] Dogan NO , Suadiye E , Wrede P , et al. Immune cell‐based microrobots for remote magnetic actuation, antitumor activity, and medical imaging. Adv Healthc Mater. 2024;13(23):2400711. 10.1002/adhm.202400711 38885528

[111] Kim DH , Rozhkova EA , Ulasov IV , et al. Biofunctionalized magnetic‐vortex microdiscs for targeted cancer‐cell destruction. Nat Mater. 2010;9(2):165‐171. 10.1038/nmat2591 PMC2810356 19946279

[112] Shen Y , Zhang W , Li G , et al. Adaptive control of Nanomotor swarms for magnetic‐field‐programmed cancer cell destruction. ACS Nano. 2021;15(12):20020‐20031. 10.1021/acsnano.1c07615 34807565

[113] Lopez S , Hallali N , Lalatonne Y , et al. Magneto‐mechanical destruction of cancer‐associated fibroblasts using ultra‐small iron oxide nanoparticles and low frequency rotating magnetic fields. Nanoscale Adv. 2022;4(2):421‐436. 10.1039/d1na00474c PMC9417452 36132704

[114] He T , Xu H , Zhang Y , et al. Glucose oxidase‐instructed traceable self‐oxygenation/hyperthermia dually enhanced cancer starvation therapy. Theranostics. 2020;10(4):1544‐1554. 10.7150/thno.40439 PMC6993236 32042321

[115] Shao F , Wu Y , Tian Z , Liu S . Biomimetic nanoreactor for targeted cancer starvation therapy and cascade amplificated chemotherapy. Biomaterials. 2021;274:120869. 10.1016/j.biomaterials.2021.120869 33984636

[116] Wu Z , Troll J , Jeong H‐H , et al. A swarm of slippery micropropellers penetrates the vitreous body of the eye. Sci Adv. 2018;4(11):eaat4388. 10.1126/sciadv.aat4388 PMC6214640 30406201

[117] Jeon S , Kim S , Ha S , et al. Magnetically actuated microrobots as a platform for stem cell transplantation. Sci Rob. 2019;4(30):eaav4317. 10.1126/scirobotics.aav4317 33137727

[118] Go G , Jeong S‐G , Yoo A , et al. Human adipose‐derived mesenchymal stem cell‐based medical microrobot system for knee cartilage regeneration in vivo. Sci Robot. 2020;5(38):eaay6626. 10.1126/scirobotics.aay6626 33022593

[119] Kim E , Jeon S , An HK , et al. A magnetically actuated microrobot for targeted neural cell delivery and selective connection of neural networks. Sci Adv. 2020;6(39):eabb5696. 10.1126/sciadv.abb5696 PMC7518876 32978164

[120] Hong J , Wu D , Wang H , et al. Magnetic fibrin nanofiber hydrogel delivering iron oxide magnetic nanoparticles promotes peripheral nerve regeneration. Regen Biomater. 2024;11:rbae075. 10.1093/rb/rbae075 PMC11272175 39055306

[121] Hwang G , Paula AJ , Hunter EE , et al. Catalytic antimicrobial robots for biofilm eradication. Sci Robot. 2019;4(29):eaaw2388. 10.1126/scirobotics.aaw2388 PMC6748647 31531409

